# Transcriptional Profiling at Single‐Cell Resolution Reveals Diversity and Regulatory Networks of Primary and Secondary Senescent Cells

**DOI:** 10.1111/acel.70540

**Published:** 2026-05-18

**Authors:** Dong‐Hyun Jang, Eunha Shim, Ji‐Won Shin, Seokho Kim, Serban Ciotlos, Hyun Jung Kim, Tae‐Hwan Gil, Yumin Kim, Ok Hee Jeon

**Affiliations:** ^1^ Department of Biomedical Sciences Department of Convergence Medicine Korea University College of Medicine Seoul Republic of Korea; ^2^ Animal Biotechnology and Genomics Division National Institute of Animal Science, Rural Development Administration Wanju Republic of Korea; ^3^ Buck Institute for Research on Aging Novato California USA; ^4^ Department of Anatomy Korea University College of Medicine Seoul Republic of Korea; ^5^ Department of Biomedical Science and Engineering Gwangju Institute of Science and Technology Gwangju Republic of Korea

**Keywords:** cellular senescence, primary senescence, SASP, secondary senescence, single‐cell RNA sequencing, transcriptional heterogeneity

## Abstract

Senescent cells accumulate with age following stress‐induced cell cycle arrest triggered by DNA damage, oncogene activation, and replicative exhaustion. While they contribute to tissue repair and tumor suppression, their persistent senescence‐associated secretory phenotypes (SASPs) drive age‐related diseases. The heterogeneity of senescent cell populations, particularly the distinction between primary and secondary senescence, remains incompletely understood at single‐cell resolution. Here, we established models of primary senescence by X‐ray irradiation of human renal epithelial cells and secondary senescence by exposing proliferating cells to conditioned media from primary senescent cells. Single‐cell RNA sequencing revealed structured transcriptional trajectories culminating in distinct terminal clusters in primary (C5, C6, and C8) and secondary (C3, C5, and C7) senescence. Primary senescence preferentially converged on extracellular matrix‐ and fibrosis‐associated programs, whereas secondary senescence exhibited more inflammatory and signaling‐responsive programs, while both contexts shared a partially overlapping transcriptional module enriched in stress‐response and cytokine‐related transcriptional modules. We identified subtype‐associated genes distinguishing primary from secondary senescent cells, as well as candidate transcriptional regulators—such as HMGA1, NFKB1, and JUNB—associated with conserved and context‐specific senescence programs. This study provides a single‐cell‐resolved transcriptional map of divergent and shared molecular features relevant to renal aging and disease.

## Graphical abstract

The research article “Transcriptional Profiling at Single‐cell Resolution Reveals Diversity and Regulatory Networks of Primary and Secondary Senescent Cells” by Jang, Shim, et al. models primary senescence by X‐ray irradiation and secondary senescence by conditioned media transfer in human renal epithelial cells. Single‐cell RNA‐seq identifies distinct terminal clusters (primary: C5, C6, and C8; secondary: C3, C5, and C7), with ECM/fibrosis programs enriched in primary cluster C8 and inflammatory/signaling programs enriched in secondary cluster C3. A shared stress/cytokine module highlights conserved and context‐specific senescence networks.

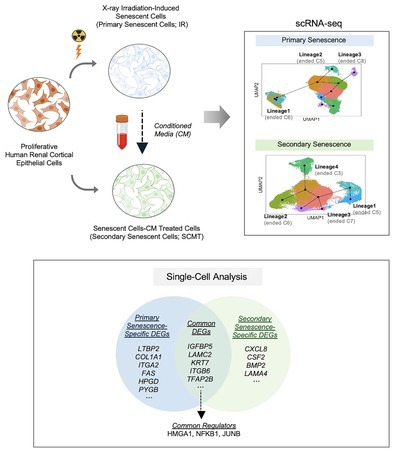

**Abbreviations**
CKD: chronic kidney disease
CM: conditioned media
DEGs: differentially expressed genes
DMEM: Dulbecco's modified Eagle medium
ECM: extracellular matrix
EdU: 5‐ethynyl‐2′‐deoxyuridine
ELISA: enzyme‐linked immunosorbent assay
ER: endoplasmic reticulum
ES: enrichment scores
FBS: fetal bovine serum
FDR: false discovery rate
GO: gene ontology
GSEA: gene set enrichment analysis
GSVA: gene set variation analysis
HVGs: highly variable genes
IR: X‐ray irradiation‐induced senescent cells
KEGG: Kyoto Encyclopedia of Genes and Genomes
KPMP: kidney precision medicine project
lncRNAs: long noncoding RNAs
log_2_FC: log_2_ fold change
NES: normalized enrichment score
QCMT: cells treated with CM from quiescent cells
QUI: quiescent cells
SASPs: senescence‐associated secretory phenotypes
SA‐β‐gal: senescence‐associated β‐galactosidase
SCENIC: single‐cell regulatory network inference and clustering
SCMT: cells treated with CM from senescent cells
scRNA‐seq: single‐cell RNA sequencing
SEM: standard error of the mean
SnCs: senescent cells
TFs: transcription factors
UMAP: uniform manifold approximation and projection
UMIs: unique molecular identifiers

## 1 Introduction

Cellular senescence is a heterogeneous process characterized by irreversible cell proliferation arrest in response to diverse stressors, including DNA damage, oncogene activation, and replicative exhaustion (Kumari and Jat 2021). Although senescence plays a protective role in early life by suppressing tumorigenesis and promoting wound healing, its prolonged presence contributes to age‐related pathologies (Van Deursen 2014). The accumulation of senescent cells (SnCs) contributes to chronic inflammation, tissue remodeling, and the progression of age‐related diseases such as fibrosis and cancer (da Silva et al. 2019; Zhang et al. 2022). These detrimental effects are largely mediated by the senescence‐associated secretory phenotype (SASP), a complex pro‐inflammatory transcriptional and secretory profile composed of cytokines, chemokines, proteases, growth factors, and other molecules (Saul et al. 2022).

A central challenge in senescence research is the inherent heterogeneity within SnC populations (Cohn et al. 2023; Hernandez‐Segura et al. 2017; Wechter et al. 2023). This heterogeneity complicates their molecular characterization and may limit the efficacy of senolytic therapies designed to selectively eliminate harmful SnCs (Gasek et al. 2021). SnCs are often classified into primary and secondary subtypes based on their mode of induction, molecular programs, and biological functions. Primary SnCs are induced directly by intrinsic stressors, such as DNA damage, whereas secondary SnCs arise through paracrine (Acosta et al. 2013; Nelson et al. 2012) or systemic signaling mechanisms driven by SASP factors released by primary SnCs (Jeon et al. 2022). This process, commonly referred to as senescence transmission or secondary senescence, enables the propagation of senescence‐associated phenotypes to neighboring cells, amplifying its impact on tissue homeostasis.

Understanding the distinction between primary and secondary SnCs is critical, as they exhibit unique transcriptomic profiles, functional behaviors, and capacities for propagating the senescence phenotype. Emerging evidence suggests that secondary senescence is regulated by pathways involving receptors for VEGF (VEGFR2, for vascular endothelial growth factor), TGF‐β (TGFBR1, for transforming growth factor β), and MCP1 (CCR2, for monocyte chemoattractant protein 1) (Acosta et al. 2013). However, as the activating ligands were not tested, it remains unclear whether these pathways serve as primary drivers or downstream responses to secondary senescence. Notably, secondary senescence exhibits stable growth arrest and SASP secretion while showing more spatially restricted propagation compared with primary senescence (Acosta et al. 2013), suggesting potential differences in tissue‐level impact.

Despite growing recognition of SnC heterogeneity, most studies have primarily focused on bulk analyses or primary senescence induced by specific stressors such as ionizing radiation or oncogene activation (Hernandez‐Segura et al. 2017). However, the transcriptional heterogeneity and dynamic progression of primary and secondary SnCs at the single‐cell level remain largely underexplored (Cohn et al. 2023). A detailed dissection of the molecular pathways and regulatory networks that distinguish these subtypes may improve our understanding of senescence biology and its contribution to age‐associated diseases.

In this study, we employed single‐cell RNA sequencing (scRNA‐seq) to dissect the transcriptional landscape of primary and secondary SnCs in human renal epithelial cells. Primary senescence was induced via X‐ray irradiation, whereas secondary senescence was modeled by exposing proliferating cells to SASP‐enriched conditioned media (CM) derived from primary SnCs. Our results revealed distinct transcriptomic signatures, lineage trajectories, and regulatory networks defining primary and secondary SnCs. Pseudotime reconstruction identified dynamic transitions from proliferative to terminal senescent states, highlighting intermediate states as potential transition phases within senescence progression. Additionally, regulatory network inference identified transcription factors (TFs), such as HMGA1, NFKB1, and JUNB, as candidate regulators associated with senescence‐associated programs.

These findings provide a single‐cell–resolved framework for distinguishing primary and secondary senescence for understanding their shared and divergent molecular mechanisms in the context of senescence‐associated renal aging and diseases.

## 2 Results

### 2.1 Single‐Cell RNA Sequencing Reveals Transcriptional Heterogeneity and Progression States in Primary Epithelial Cell Senescence

**FIGURE 1:**
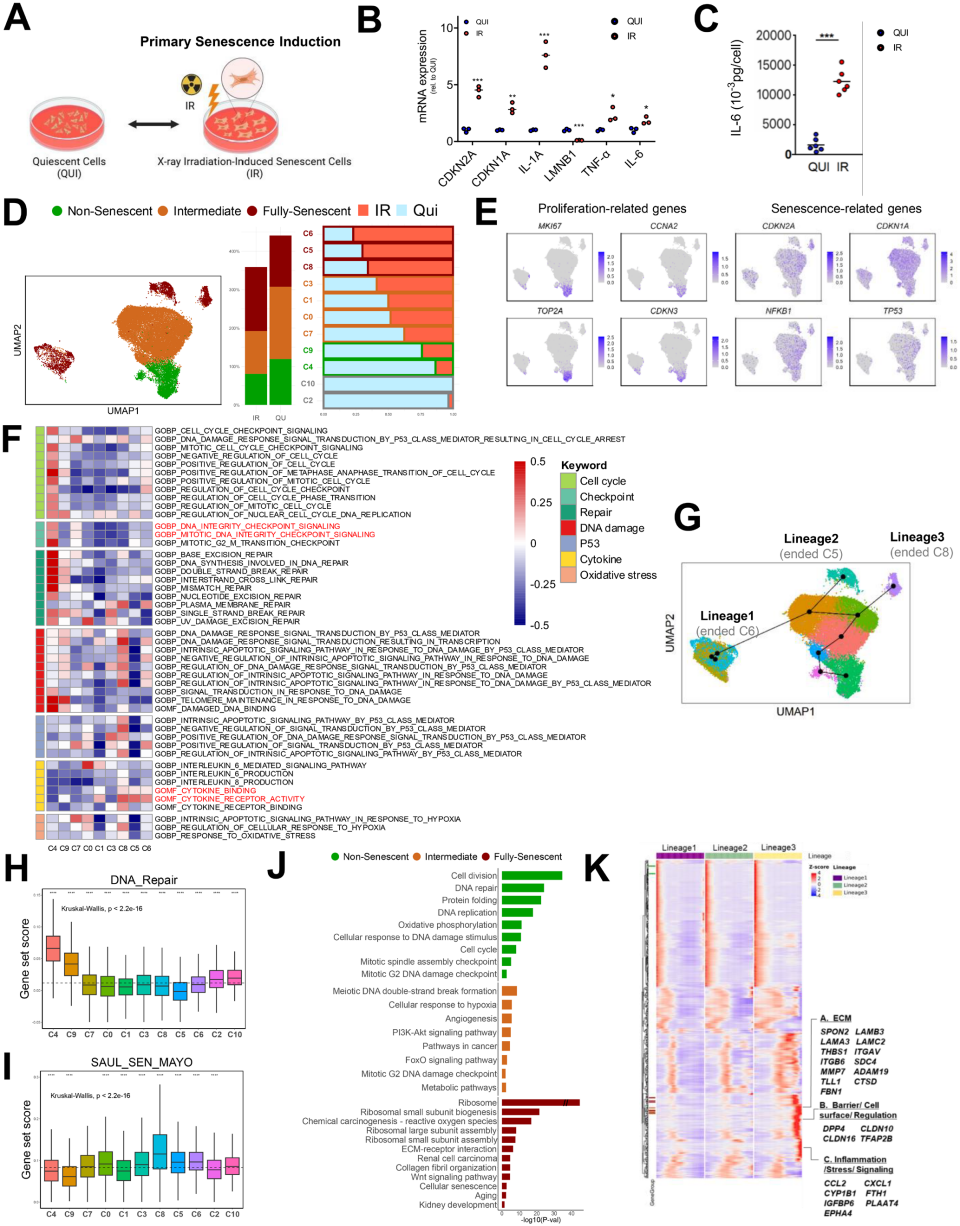
Transcriptional heterogeneity and lineage‐resolved progression in primary senescence at single‐cell level. (A) Experimental overview. Renal epithelial cells were irradiated (IR; 10 Gy, 10 days) to induce primary senescence, with quiescent controls (QUI; 0.01% serum, 3 days) processed for scRNA‐seq. (B) Expression levels of senescence and SASP‐related genes in senescent relative to the controls (QUI, *n* = 3; IR, *n* = 3). (C) Secreted IL‐6 levels in CM measured using ELISA (QUI, *n* = 6; IR, *n* = 6). Data are presented as the means ± the standard error of the mean (unpaired two‐tailed *t*‐test; *p < 0.05, **p < 0.01, ***p < 0.001). (D) UMAP of primary dataset showing clusters grouped into non‐senescent (C4 and C9), intermediate (C0, C1, C3, and C7), and fully senescent states (C5, C6, and C8) (left). Each bar represents either IR or QUI, and each colored segment's height indicates the fraction of one of the three senescence states within that group (middle). Stacked bar chart showing the proportions of IR and QUI cells across each cluster (right). (E) Feature plots showing expression levels of proliferation and senescence‐associated genes. (F) Heatmap of pathway activity across clusters scored via gene set variation analysis, with *Z*‐score normalization. (G) UMAP trajectory analysis using Slingshot identifying three senescence progression lineages. Trajectory lines overlaid on UMAP. Cell clusters are colored by pseudotime progression. (H, I) Boxplots of normalized pathway scores for DNA repair (H) and SASP‐related gene sets (I) across clusters (Kruskal–Wallis test, with pairwise Wilcoxon rank‐sum test; adjusted *p‐values* as shown). (J) Enriched pathways of non‐senescent, intermediate, and fully senescent states in the primary SnCs. *p‐values* were calculated using a hypergeometric distribution. (K) TradeSeq‐based heatmap of temporally regulated top 500 genes along the pseudotime trajectory for lineage 3 (*p* < 0.05), with representative late‐pseudotime genes highlighted.

Cellular senescence advances through diverse phenotypic states following growth arrest. To dissect these states in renal epithelial cells, we performed scRNA‐seq after inducing primary senescence by 10 Gy X‐irradiation. Irradiated cells (IR) were cultured for 10 days posttreatment, while quiescent controls (QUI) were generated by serum reduction (0.01% serum) for 3 days (Figure 1A). Senescence induction was validated by increased senescence‐associated β‐galactosidase (SA‐β‐gal) activity, reduced 5‐ethynyl‐2′‐deoxyuridine (EdU) (+) proliferative cells, upregulation of senescence/SASP‐associated genes (*CDKN2A*, *CDKN1A*, *IL‐1A*, *TNF‐α*, and *IL‐6*), reduced *LMNB1* expression, and increased secretion of the SASP factor IL‐6 into the CM (Figure 1B,C and Figure S1A–C).

Unsupervised clustering revealed eleven transcriptional clusters (Figure S1D). C2 and C10, primarily composed of QUI cells (> 90%), were excluded from subsequent analyses to focus on clusters representing senescence progression. Uniform manifold approximation and projection (UMAP) analysis (Figure 1D), along with cell cycle scoring analysis (Figure S1E) categorized the remaining clusters into three major cell states: non‐senescent (C4 and C9), intermediate (C0, C1, C3, and C7), and fully senescent (C5, C6, and C8). Non‐senescent clusters expressed canonical proliferation genes such as *MKI67*, *CCNA2*, *TOP2A*, and *CDKN3*, whereas intermediate clusters displayed mixed proliferative and senescent signatures. Fully senescent clusters were dominated by IR cells and exhibited strong upregulation of senescence‐related genes (e.g., *CDKN2A*, *CDKN1A*, and *TP53*) and SASP factors, such as *NFKB1*, *SERPINE1*, *SERPINE2*, *TNF*, and *IL‐6* (Figure 1E and Table S1).

Gene set variation analysis (GSVA) further showed progressive activation of senescence‐related gene sets across intermediate clusters (C0, C1, C3, and C7) and emphasized functional differences among fully senescent clusters (C5, C6, and C8) (Figure 1F and Figure S1F). For example, C5 was enriched in genes related to kidney development, whereas C6 and C8 exhibited increased activity in stress‐response programs, including DNA damage response and apoptosis‐associated gene sets. Across all fully senescent clusters, gene sets for cytokine signaling and receptor activity were consistently elevated, indicating a shared inflammatory component despite subtype heterogeneity.

To infer senescence progression within this single post‐induction timepoint, we reconstructed trajectories using Slingshot (Figure 1G). Three lineages were identified: (i) one terminating in C5, characterized by DNA damage‐associated signatures; (ii) ending in C6, marked by nucleolar stress and ribosomal activity; (iii) culminating in C8, enriched in SASP‐related inflammatory markers. Gene set scoring supported a stepwise transition from non‐senescent to fully senescent states, marked by decreasing DNA repair and increasing SASP‐related signatures (Figure 1H,I and Figure S1G). We next defined senescence stage‐associated transcriptional programs (non‐senescent, intermediate, and fully senescent) and summarized their biological pathways (Figure 1J and Table S2). Non‐senescent states were enriched in cell division and DNA replication pathways, while intermediate states activated mitotic G2 DNA damage checkpoint and meiotic DNA double‐strand breaks, signifying early senescence commitment. Fully senescent states displayed upregulation of ribosomal activity, extracellular matrix (ECM)‐receptor interaction, and oxidative stress‐linked programs.

Finally, tradeSeq analysis along lineage 3 ending in C8, identified temporally changed gene modules across pseudotime to lead to fully senescent states. Early pseudotime was dominated by proliferative and DNA repair genes (*UBE2C*, *TOP2A*, and *MKI67*), followed by stress‐associated intermediate signatures (*SAA2*, *SAA1*, *LINC01446*, *IGFBP2*, and *TUBB2B*), and late pseudotime exhibited strong enrichment for ECM remodeling/adhesion and inflammatory programs. Late‐pseudotime genes in C8 included ECM/remodeling genes (e.g., SPON2, *LAMB3*, *LAMA3*, *LAMC2*, *THBS1*, *ITGAV*, *ITGB6*, *SDC4*, *MMP7*, *ADAM19*, *TLL1*, *CTSD*, and *FBN1*), barrier/cell‐surface genes (*DPP4*, *CLDN10*, *CLDN16*, and *TFAP2B*), and inflammation/stress‐associated genes (*CCL2*, *CXCL1*, *CYP1B1*, *FTH1*, *IGFBP6*, *PLAAT4*, and *EPHA4*) (Figure 1K, Figure S1H and Table S3), consistent with a terminal senescent program characterized by elevated SASP activity and tissue remodeling features.

These findings resolve non‐senescent, intermediate, and fully senescent states in irradiation‐induced primary senescence and define lineage‐dependent terminal programs, with C8 representing a prominent SASP‐high, ECM‐remodeling–enriched endpoint.

### 2.2 Transcriptional Heterogeneity and Cellular States of SASP‐Related Secondary Senescence

**FIGURE 2:**
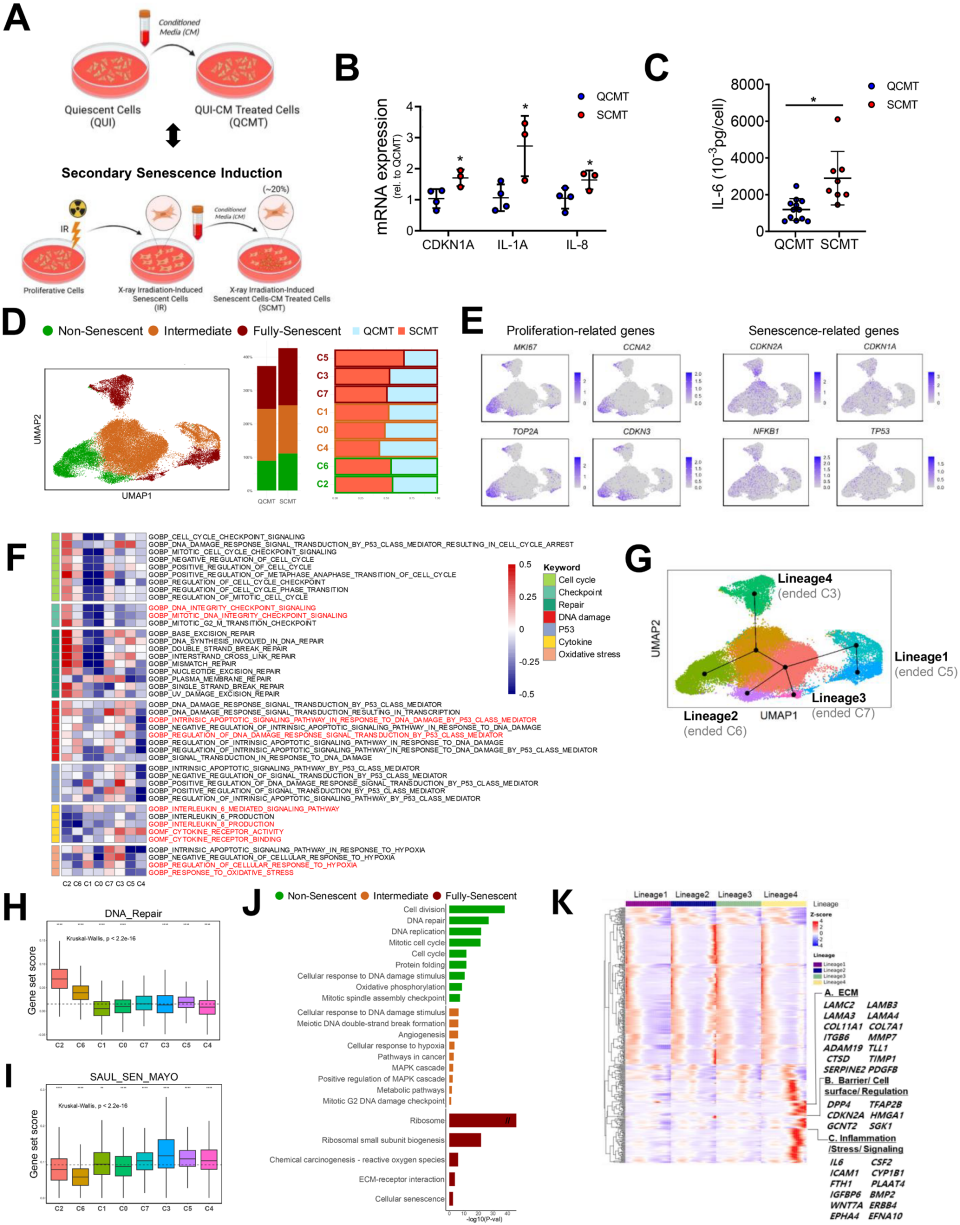
SASP‐driven secondary senescence shows distinct transcriptional states. (A) Experimental overview: Proliferative renal epithelial cells were treated with CM from quiescent cells (QCMT) or primary senescent cells (SCMT) and separately processed for scRNA‐seq. (B) qPCR validation of senescence/SASP‐associated genes and expressed as fold changes in SCMT versus QCMT (QCMT, *n* = 4; SCMT, *n* = 3). Data are presented as the mean ± standard error of the mean. *p < 0.05, **p < 0.01, ***p < 0.001 (two‐tailed unpaired *t*‐test) (C) Secreted IL‐6 levels in CM measured by ELISA (QCMT, *n* = 12; SCMT, *n* = 8). (D) UMAP of secondary SnCs showing clusters grouped into non‐senescent (C2 and C6), intermediate (C0, C1, and C4), and fully senescent clusters (C3, C5, and C7) (left). Each bar represents either QCMT or SCMT, and each colored segment's height indicates the fraction of one of the three senescence states within that group (middle). Stacked bar chart showing the proportions of QCMT and SCMT cells across each cluster (right). (E) Feature plots of representative proliferation and senescence‐associated genes across clusters. (F) Heatmap of pathway activities across clusters (*Z*‐score normalized). (G) UMAP trajectory analysis using Slingshot identifies four lineages with distinct terminal clusters, including a senescence‐resistant endpoint. Trajectory lines indicate senescence progression, and clusters are colored by pseudotime. (H, I) Boxplots of DNA repair (H) and SASP‐related gene set scores (I) across clusters (Kruskal–Wallis two‐sided test with pairwise Wilcoxon rank‐sum test; adjusted *p‐values* as shown). (J) Enriched pathways categorized into non‐senescent, intermediate, and fully senescent states. *p‐values* were calculated using a hypergeometric distribution. (K) Heatmap displaying temporally regulated the top 500 genes identified through tradeSeq along the pseudotime trajectory for lineage 4 in secondary senescence (hypergeometric distribution; *p* < 0.05).

To model secondary senescence driven by paracrine cues, proliferating renal epithelial cells were treated with CM from primary SnCs (SCMT), while control cells were treated with CM from quiescent cells (QCMT) (Figure 2A). Secondary senescence induction was validated by increased SA‐β‐gal (+) cells, reduced EdU (+) proliferative cells, reduced number of cells without nuclear HMGB1, upregulation of senescence‐associated genes (*CDKN1A*, *IL‐1A*, and *IL‐8*), and elevated IL‐6 secretion (Figure 2B,C and Figure S2A–D), consistent with the acquisition of an inflammatory signature through SASP‐mediated signaling.

scRNA‐seq analysis identified eight transcriptionally distinct clusters (Figure S2E), which were classified into three major states: non‐senescent (C2 and C6), intermediate (C0, C1, and C4), and fully senescent (C3, C5, and C7) (Figure 2D). Differentially expressed gene (DEG) analysis showed that non‐senescent clusters retained cell cycle‐related markers and pathway analysis suggested functional divergence even within the non‐senescent compartment. C2 showed upregulated DNA damage checkpoint and repair pathways, whereas C6 lacked this response, consistent with a comparatively senescence‐resistant profile (Figure 2E and Table S4). Intermediate clusters (C0, C1, and C4) exhibited mixed proliferative and senescence‐associated signatures, suggesting a transitional identity. Unlike C0 and C1, showing genes related to immune and inflammatory responses along with partial activation of pathways typically triggered by stress factors (e.g., oxidative stress, hypoxia, or DNA damage), C4 displayed the activation of pathways related to senescence and cancer signaling as well as upregulation of both DNA damage/repair mechanisms (Table S2). This suggests that C4 is a cluster containing transitional cells that could be linked to both senescence and cancer development. Fully senescent clusters (C3, C5, and C7) upregulated senescence markers (*CDKN2A*, *CDKN1A*, and *TP53*) and SASP factors *NFKB1* and *IL‐6* (Figure 2E and Table S1), corroborated by cell cycle analysis (Figure S2F).

GSVA further distinguished functional programs across clusters (Figure 2F and Figure S2G). Non‐senescent clusters were enriched in cell cycle and DNA repair pathways, with C2 showing activation of DNA integrity checkpoint signaling, while C6 lacked this response, reinforcing its senescence‐resistant phenotype. While fully senescent clusters shared cytokine receptor activity and DNA damage‐associated signatures, they also exhibited subtype differences. C3 was enriched for p53‐related gene sets and IL‐8 production, C5 showed IL‐6‐mediated signaling, and C7 exhibited hypoxia‐related gene set enrichment, emphasizing heterogeneity among secondary senescent states. Notably, compared with primary senescent clusters (C5, C6, and C8 in Figure 1F and Figure S1F), secondary senescent clusters (C3, C5, and C7) retained relatively higher DNA repair activity and did not show enrichment of renal carcinoma–linked gene sets (Figure 2F and Figure S2G).

Pseudotime trajectory analysis using Slingshot revealed four lineages leading to distinct terminal states (C3, C5, C6, and C7) (Figure 2G). The lineage terminating in C6 corresponded to the senescence‐resistant trajectory and was excluded from downstream terminal‐senescence program interpretation. Gene set scoring along pseudotime supported progressive DNA repair loss and increased SASP‐associated signatures toward fully senescent clusters (Figure 2H,I and Figure S2H). Stage‐associated pathway patterns toward fully senescent states during secondary senescence were shown in Figure 2J to highlight a transition from cell cycle and DNA repair programs to checkpoint/stress‐associated programs (e.g., mitotic G2 DNA damage checkpoint activation, meiotic DNA double‐strand breaks, and the MAPK cascade) and ultimately to fully senescent states, including upregulated ribosomal activity, ECM‐receptor interactions, and oxidative stress–linked pathways (Figure 2J and Table S2).

TradeSeq analysis focusing on lineage 4 (ending in C3) revealed a dynamic transcriptional shift with enrichment of cell adhesion/migration‐related pathways (GO), PI3K–Akt signaling and focal adhesion (KEGG), and ECM organization and receptor tyrosine kinase signaling (Reactome) (Figure S2I and Table S3). Compared to primary senescence lineage 3 (ending in C8), which was dominated by ECM–receptor interaction and ECM organization/turnover, secondary lineage 4 additionally showed stronger enrichment of growth factor–responsive pathways alongside inflammatory/SASP‐associated components, including *IL6* and *CSF2* (Figure 2K).

These findings demonstrate that SASP‐conditioned media induce heterogeneous secondary senescent states with distinct terminal trajectories, including inflammation/SASP‐associated, an IL‐6–linked signaling trajectory accompanied by IFN‐α, DNA‐damage, and broader interleukin programs, a p53/ribosome‐associated stress‐adaptation trajectory enriched for hypoxia responses, and a senescence‐resistant trajectory.

### 2.3 Primary and Secondary Senescence Exhibit Distinct Yet Partially Overlapping Transcriptional Programs

**FIGURE 3:**
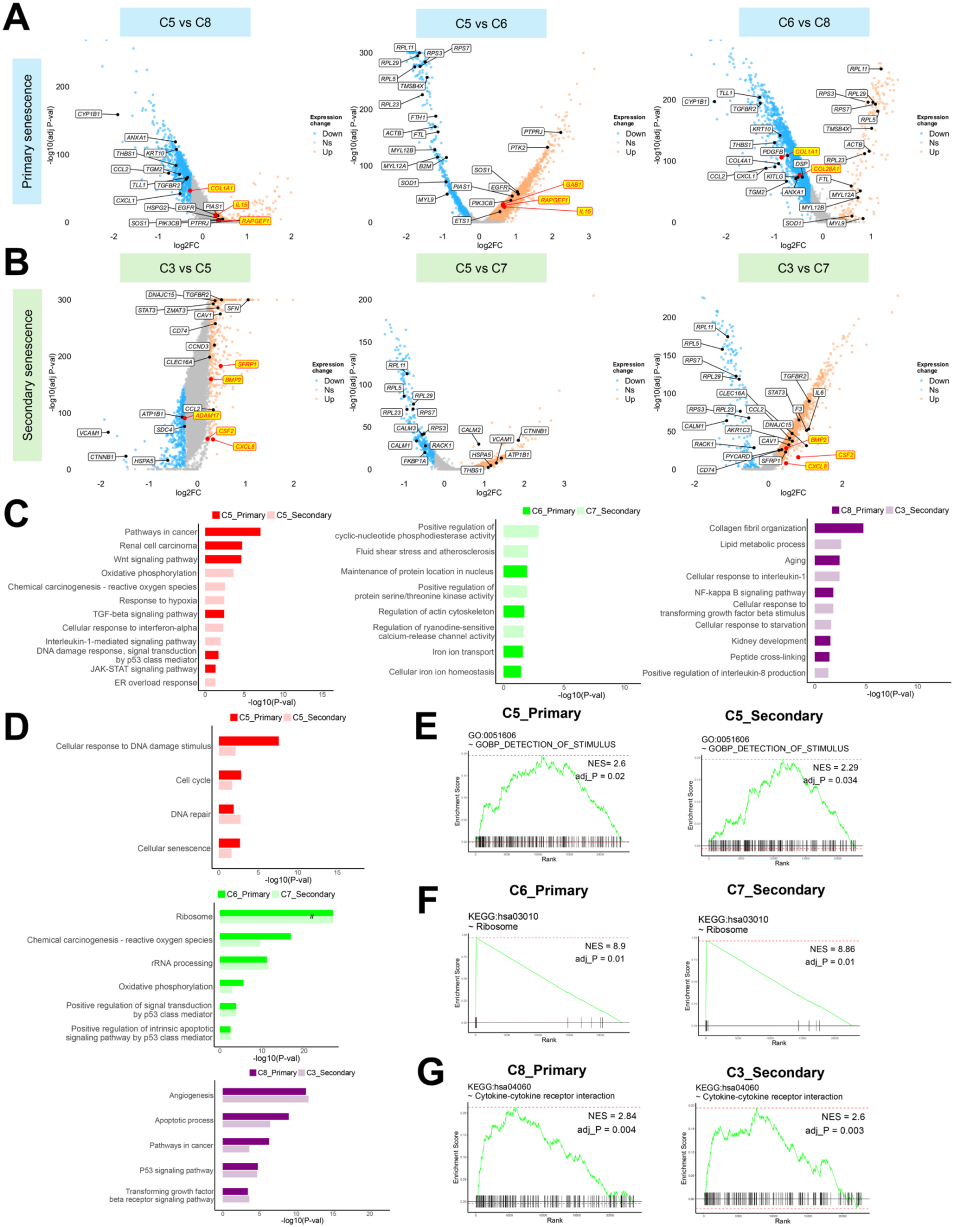
Distinct and shared transcriptional programs in primary and secondary senescence. (A) Volcano plots of DEGs between primary senescent clusters (C5 vs. C8 (left), C5 vs. C6 (middle), and C6 vs. C8 (right)). (B) Volcano plots of DEGs in secondary senescent clusters (C3 vs. C5 (left), C5 vs. C7 (middle), and C3 vs. C7 (right)). The x‐axis represents the log_2_ fold change (log_2_FC), and the y‐axis shows the −log_10_ (adjusted *p*‐values). Upregulated genes are indicated in orange, downregulated genes in blue. Selected genes are labeled, with cluster‐specific genes highlighted in red text with a yellow background. DEGs were identified using the Wilcoxon Rank‐Sum test in Seurat with Bonferroni‐ adjusted *p‐value*s < 0.05, log_2_FC > 0.25. (C, D) Pathway enrichment analysis of senescence‐specific (C) and shared (D) pathways in primary and secondary senescent clusters. The hypergeometric distribution was used to calculate *p‐value*s. (E–G) GSEA showing pathways significantly enriched in corresponding primary and secondary senescent clusters. Enrichment was determined using a one‐sided permutation test, with the normalized enrichment score (NES) and false discovery rate (FDR) controlled using the Benjamini–Hochberg procedure. Running enrichment scores (ES), NES, and adjusted *p‐value*s are shown for each pathway.

Building on the lineage‐resolved states identified in Figures 1 and 2, we next directly compared senescent clusters from primary (C5, C6, and C8) and secondary (C3, C5, and C7) datasets to determine whether the terminal trajectories inferred by pseudotime analysis were reflected in cluster‐level transcriptional architecture (Figure 3A,B).

Within primary senescence, each terminal cluster displayed a distinct transcriptional profile consistent with its lineage identity. C5 exhibited upregulated JAK–STAT signaling (e.g., *IL15*, *EGFR*, and *PIAS1*), renal cell carcinoma (e.g., *RAPGEF1*, *SOS1*, *PIK3B*, *GAB1*, and *ETS1*), and cell growth‐regulatory signaling components (e.g., *PTK2*, *HSPG2*, and *PTPRJ*), whereas C6 showed increased expression of ribosomal and nucleolar stress‐related genes (e.g., *RPS3*, *RPL29*, *RPL5*, *RPL11*, etc.) (Lessard et al. 2018; Mauvezin et al. 2020), oxidative stress (*SOD1* and *B2M*) and iron metabolism (*FTL* and *FTH1*), reinforcing a distinct metabolic profile (Figure 3A). In contrast, C8 showed strong ECM remodeling activity, including collagen family members and matrix‐remodeling factors (e.g., *COL28A1*, *COL1A1*, *COL4A1*, *THBS1*, and *TGM2*), consistent with the ECM‐dominant terminal program observed in lineage 3 of primary senescence (Figure 1K).

In secondary senescence, C3 showed a pronounced inflammatory/SASP‐associated profile, marked by *CXCL8*, *BMP2*, *CSF2*, *IL‐6*, *STAT3*, and *PYCARD* genes (Figure 3B). C5 showed the upregulation of genes involved in stress‐and endoplasmic reticulum (ER)‐associated signaling components (*HSPA5*), while C7 demonstrated a ribosome‐ and calcium signaling–associated signature, involving *CALM1*, *CALM3*, and *FKBP1A*, partially overlapping with the nucleolar stress–like features of primary C6.

Pathway analysis further highlighted this functional divergence (Figure 3C and Table S2). C5 (primary) was enriched in cancer‐associated pathways, such as the renal cell carcinoma, Wnt signaling pathway, and the JAK–STAT signaling pathway, whereas C5 (secondary) increased oxidative stress and inflammation‐related pathways (e.g., cellular response to interferon‐α and interleukin‐1‐mediated signaling pathway). C6 (primary) was enriched for iron homeostasis, whereas C7 (secondary) was enriched in calcium signaling and ion channel regulation. Another striking difference was observed in C8 (primary), which was highly enriched in ECM remodeling pathways (e.g., collagen fibril organization and peptide crosslinking), reinforcing its fibrosis role, whereas C3 (secondary) preferentially enriched in lipid metabolism, cellular response to starvation, and cytokine‐production pathways (e.g., cellular response to TGF‐β stimulus and interleukin‐8 production), consistent with an inflammatory/SASP‐associated phenotype.

Despite these distinctions, several pathways were shared across corresponding clusters (Figure 3D). C5 in both primary and secondary senescence exhibited upregulation of DNA damage response, cell cycle, and DNA repair pathways, underscoring their role in senescence maintenance. Similarly, C6 (primary) and C7 (secondary) shared ribosome‐related signaling, suggesting partially conserved nucleolar stress responses across senescence contexts, while C8 (primary) and C3 (secondary) were enriched in apoptotic and angiogenesis‐related pathways.

Gene set enrichment analysis (GSEA) further reinforced these findings. In both primary and secondary senescence, C5 was enriched in stimulus detection, a hallmark of cellular senescence (Figure 3E). C6 (primary) and C7 (secondary) uniquely displayed upregulation of ribosome‐related pathways, consistent with their nucleolar stress‐mediated transcriptional profiles (Figure 3F). C8 (primary) and C3 (secondary) were strongly enriched in cytokine–cytokine receptor interactions, underscoring a shared inflammatory component (Figure 3G).

Collectively, these analyses demonstrate that while primary and secondary senescence share core stress‐response, ribosomal activity, and cytokine–cytokine receptor interaction, their terminal transcriptional and functional programs diverge in a context‐dependent manner. Primary senescence is characterized by a matrix‐remodeling–dominant program, whereas secondary senescence exhibits a more inflammatory and signaling‐responsive phenotype.

### 2.4 Identification and Validation of Subtype‐Specific Genes in Primary and Secondary Senescence

Building on the lineage‐resolved and cluster‐level divergence shown in Figures 1, 2, 3, we next sought to identify representative molecular genes that distinguish primary and secondary senescent subtypes.

**FIGURE 4:**
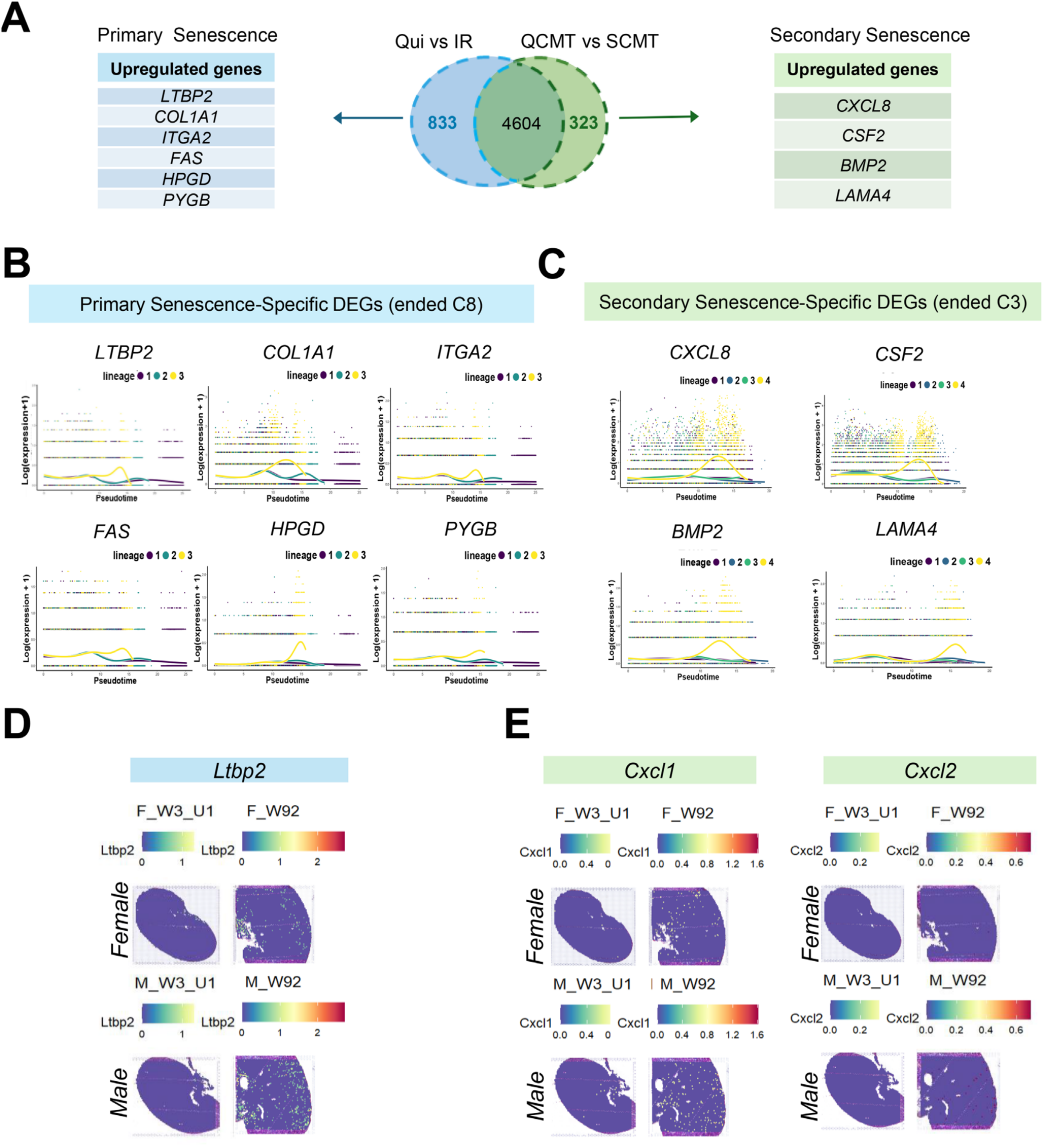
Identification and in vivo validation of subtype‐specific genes in primary and secondary senescence. (A) Venn diagram illustrating overlapping and unique DEGs between primary (QUI vs. IR) and secondary (QCMT vs. SCMT) senescence (adjusted *p‐value*s < 0.05, log_2_FC > 0.25). Upregulated genes specific to each condition are listed. (B) Pseudotemporal expression profiles of representative primary senescence–specific DEGs enriched in the terminal primary cluster (C8). The x‐axis represents the pseudotime and the y‐axis represents the log‐normalized gene expression levels. (C) Pseudotime expression profiles of representative secondary senescence–specific DEGs enriched in the terminal secondary cluster (C3). Gene expression dynamics are shown along lineage 4 toward the terminal senescent state. (D, E) Spatial transcriptomic validation using a public mouse kidney Visium dataset (GSE252772). (D) Spatial expression of *Ltbp2*, a primary senescence–specific DEG and (E) *Cxcl1*/*Cxcl2* (murine homologs of human *CXCL8*; secondary senescence–specific DEG) by comparing young (W3) and aged (W92) kidneys. Spatial feature maps were generated from SCTransform‐normalized Visium data merged into a single Seurat object and batch‐corrected with Harmony using sample_id. F, female; M, male; W, week; U1 indicates an individual Visium dataset identifier. The color scale represents normalized gene expression level.

Comparison of DEGs between the primary senescence (QUI vs. IR) and secondary (QCMT vs. SCMT) senescence datasets identified 833 primary‐specific and 323 secondary‐specific genes (Figure 4A and Tables S5 and S6). Further refinement at the cluster level distinguished primary senescent clusters (C5, C6, and C8) and secondary senescent clusters (C3, C5, and C7) from other populations, linking subtype‐enriched genes to senescence, SASP activity, and renal function (Table S7). To focus on terminal states most relevant to senescence propagation, we prioritized the senescent clusters representing lineage endpoint with the highest SASP activity—C8 in primary senescence and C3 in secondary senescence.

Primary C8 exhibited significant upregulation of genes related to ECM remodeling (*COL1A1* and *ITGA2*), apoptotic processes (*FAS*), TGF‐β signaling (*HPGD, LTBP2*), and metabolic dysregulation (*PYGB*) (Figure 4A). Pseudotime trajectory analysis confirmed progressive upregulation of these genes toward the terminal endpoint of lineage 3 (Figure 4B), consistent with the ECM‐remodeling–dominant architecture identified in Figures 1 and 3.

In contrast, secondary C3 exhibited upregulation of inflammatory/SASP‐associated genes (*CXCL8*, *CSF2*, and *BMP2*) and ECM glycoproteins (*LAMA4*) (Figure 4A) (Ros and Schulze 2012). Pseudotime analysis demonstrated progressive upregulation of these genes along lineage 4 toward the terminal secondary state (Figure 4C), supporting an inflammatory/SASP‐dominant terminal program in secondary senescence.

To evaluate the in vivo relevance of these primary‐ and secondary‐senescence–specific DEGs, we analyzed a public mouse kidney spatial transcriptomics dataset (GSE252772) comparing young (week 3) and aged (week 92) kidneys of both sexes (Project 2026). Spatial mapping revealed increased expression of *Ltbp2* in aged kidneys, consistent with its progressive induction toward terminal primary senescence observed in our pseudotime analysis. In parallel, *Cxcl1*/*Cxcl2* (murine homologs of human *CXCL8* and representative secondary senescence–specific DEGs) exhibited age‐associated increased inflammatory/secretory programs in aged renal tissue (Figure 4D,E).

We further interrogated the Kidney Precision Medicine Project (KPMP) Kidney Tissue Atlas to assess clinical relevance in human chronic kidney disease (CKD) (Figure S3). Fibrosis‐enriched regions, defined by spatial co‐expression of *ACTA2* and *COL1A1*, showed increased expression of multiple primary senescence‐associated DEGs, including *LTBP2*, compared with healthy reference tissues. Secondary senescence‐associated DEGs displayed more heterogeneous spatial enrichment, presumably due to their context‐dependent inflammatory/secretory activation.

To provide limited protein‐level assessment, we measured LTBP2 in CM and CXCL8 in cell lysates across senescence conditions (Figure S4). LTBP2 showed higher abundance in IR‐CM, whereas CXCL8 levels were elevated in SCMT lysates. Given variability across samples and compartments, these measurements are interpreted as exploratory and supportive rather than definitive validation.

Collectively, these findings reveal distinct molecular signatures underlying primary and secondary senescence. Primary senescence is characterized by a fibrosis/ECM‐remodeling–associated program (e.g., *LTBP2*), whereas secondary senescence is marked by an inflammatory/SASP‐dominant program with paracrine signaling features (e.g., *CXCL8*). Spatial transcriptomic analyses support the in vivo relevance of these subtype‐associated markers, while limited protein‐level measurements provide complementary context.

### 2.5 Shared Transcriptional Regulators and Conserved Pathways Across Primary and Secondary Senescence

**FIGURE 5:**
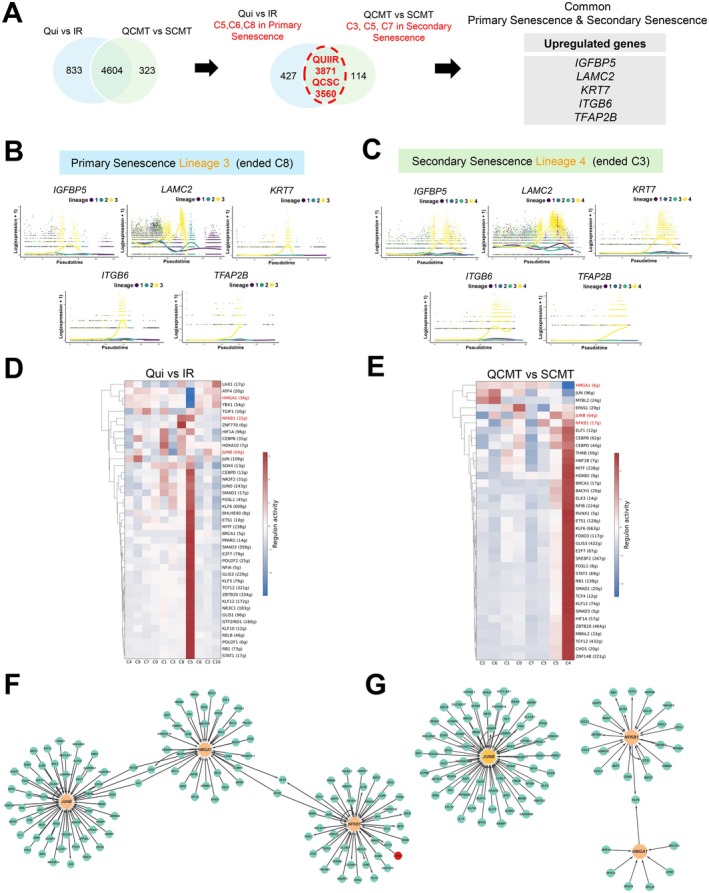
Shared transcriptional programs and regulatory networks across primary and secondary senescence. (A) Identification of shared DEGs between primary and secondary senescent clusters. Venn diagram showing overlap of DEGs between QUI vs. IR and QCMT vs. SCMT datasets (adjusted *p‐value*s < 0.05, log_2_FC > 0.25) (left). Refinement of overlapping DEGs enriched in primary senescent clusters (C5, C6, and C8) and secondary senescent clusters (C3, C5, and C7) (middle). The top five commonly upregulated genes are listed (right). (B, C) Pseudotime expression dynamics of representative shared marker genes along primary (lineage 3, ending in C8) (B) and secondary (lineage 4, ending in C3) (C) senescence trajectories. The x‐axis represents pseudotime and the y‐axis indicates log‐normalized gene expression levels. (D, E) Heatmaps displaying regulon activity of TFs identified shared or enriched as DEGs in primary senescence (D) and secondary senescence (E) clusters. Color scale represents relative regulon activity (blue, low; red, high). Thresholds: adjusted *p‐value*s < 0.05 and log_2_FC > 0.25. (F, G) SCENIC‐inferred transcriptional regulatory networks in primary (F) and secondary senescence (G). Large orange nodes represent TFs (e.g., HMGA1, NFKB1, and JUNB) and small green nodes represent predicted target genes. LAMC2, one of the commonly upregulated shared genes, is highlighted in red within the NFKB1‐associated network.

To delineate transcriptional programs conserved across primary and secondary senescent subtypes, we integrated differential expression, pseudotime dynamics, spatial validation, and transcription factor (TF) activity analyses. Comparison of senescent clusters from primary (C5, C6, and C8) and secondary (C3, C5, and C7) senescence identified 4604 overlapping DEGs (Table S8). Of these, 17 genes were consistently upregulated across both types of senescence, including *IGFBP5*, *LAMC2*, *KRT7*, *ITGB6*, and *TFAP2B* (Figure 5A and Table S9). *IGFBP5*, *KRT7*, *ITGB6*, and *TFAP2B* were predominantly enriched in terminal clusters C8 (primary) and C3 (secondary), while *LAMC2* was elevated in C8 (primary) as well as in C3 and C5 (secondary) (Figure 5B,C and Table S9). These data suggested the convergence of distinct senescence trajectories onto partially overlapping endpoint transcriptional programs.

Pseudotemporal analysis demonstrated progressive induction of these shared genes along lineage 3 (primary) and lineage 4 (secondary) (Figure 5B,C), indicating that the shared signature is associated with mature senescence rather than transitional intermediates. Functional annotation linked *LAMC2* and *ITGB6* to ECM remodeling and kidney disease progression, while *TFAP2B* was associated with apoptosis‐related pathways (Table S9), supporting the presence of a conserved stress‐ and remodeling‐associated module across senescence subtypes.

To assess clinical relevance, we interrogated spatial transcriptomic data from the KPMP Kidney Tissue Atlas (Figure S5). Common DEGs shared between primary and secondary senescence exhibited increased or spatially restricted expression in CKD samples compared with healthy reference kidneys. Notably, enrichment frequently localized to fibrotic regions defined by spatial co‐expression of *ACTA2* and *COL1A1*. These findings support the in vivo relevance of a shared senescence‐associated transcriptional module in CKD‐associated tissue remodeling.

SCENIC analysis further identified HMGA1, NFKB1, and JUNB as transcriptional regulators active in both senescent subtypes. Regulon heatmaps revealed primary‐ and secondary‐dependent activity patterns across clusters (Figure 5D,E and Table S10). HMGA1 activity was prominent in C6 (primary) and C7 (secondary), consistent with ribosomal biogenesis and metabolic signatures (Table S11). NFKB1 activity was enriched in C8 (primary) and C3 (secondary), controlled AGE‐RAGE signaling, TNF‐mediated inflammation, and ECM organization (Table S11). JUNB showed differential activation across subtypes, with higher activity in C8 (primary) and C5 (secondary) (Figure 5D,E), suggesting modulatory rather than uniformly shared regulation (Table S11).

Network visualization highlighted partially overlapping TF–target relationships (Figure 5F,G and Table S12). For example, *LAMC2*, a key fibrosis‐associated ECM remodeling gene, was predicted to be an NFKB1 target in primary senescence. Together, these analyses indicate that primary senescence preferentially adopts a matrix‐remodeling and fibrosis‐associated program, whereas secondary senescence is more strongly characterized by inflammatory and SASP‐dominant signaling. Despite these functional distinctions, both senescence contexts converge on partially overlapping and interconnected transcriptional networks that define a shared late‐stage senescence molecular signature.

## 3 Discussion

Cellular senescence is a heterogeneous and context‐dependent process that exerts both beneficial and detrimental effects across the lifespan. Although beneficial in early life for tumor suppression and wound healing, the accumulation of SnCs with aging contributes to chronic inflammation, tissue dysfunction, and fibrosis via persistent SASP secretion. While the influence of SnCs on surrounding cells is well documented (Gasek et al. 2021; Van Deursen 2014), subtype‐specific contributions of primary and secondary senescence to kidney disease and aging remain limited (Zhang et al. 2022). Importantly, most current senotherapeutic strategies target senescence broadly, overlooking the diversity within SnC populations or the potentially distinct roles of primary and secondary senescence (Sturmlechner et al. 2017).

To address this gap, we employed single‐cell transcriptomic profiling to systematically characterize primary and secondary senescence, revealing distinct lineage architectures, terminal states, and regulatory networks. Our analyses demonstrate that primary and secondary senescence are not uniform states but instead comprise structured trajectories culminating in transcriptionally and functionally distinct programs.

Primary senescence, directly induced by DNA damage from irradiation, preferentially converged on matrix remodeling and fibrosis‐associated transcriptional programs. Terminal primary senescent clusters were enriched for ECM components and remodeling factors such as *COL1A1*, *ITGA2*, and *LTBP2*, alongside pathways linked to TNF and TGF‐β signaling. These features align with fibrotic remodeling processes observed in aged and diseased kidneys. Conversely, secondary senescence, induced by SASP‐mediated paracrine signaling, exhibited more inflammatory and signaling‐responsive programs. Terminal secondary clusters were characterized by the enrichment of cytokine and immunomodulatory genes such as *CXCL8*, *CSF2*, *BMP2*, and *LAMA4*, consistent with the amplification of inflammatory microenvironments.

Pseudotime trajectory analysis further revealed that both primary and secondary senescence progress through identifiable intermediate states before reaching terminal phenotypes. Primary senescence trajectories culminated in DNA damage (C5), ribosomal stress (C6), and ECM‐remodeling and fibrotic signatures (C8). Especially, lineage 3, ending in C8, showed enrichment in ECM organization and negative regulation of cell population proliferation, suggesting that these primary SnCs actively reshape the ECM, potentially amplifying age‐related fibrosis and inflammation. These cells also secreted SASP factors, which may further amplify deleterious effects on surrounding cells (Lopes‐Paciencia et al. 2019). Secondary SnCs displayed structured transitions associated with inflammation (C3), IL‐6 signaling (C5), and ribosomal/p53 stress (C7). Secondary SnCs in lineage 4 (leading to C3) were enriched for pathways regulating cell population proliferation and PI3K‐Akt signaling. These findings support a model in which senescence represents a dynamic and progressive state transition rather than a static endpoint.

Despite their divergent transcriptional and functional programs, primary and secondary senescence converged on partially overlapping transcriptional signatures. Shared pathways including stress‐response p53 signaling, cytokine‐receptor interactions, and apoptosis‐related processes were observed in both C8 (primary) and C3 (secondary). We identified common genes such as *IGFBP5*, *LAMC2*, *KRT7*, *ITGB6*, and *TFAP2B* that were enriched in terminal clusters of both senescence types and were also spatially enriched in fibrotic regions of human CKD samples. These findings suggest that distinct senescence trajectories may converge on a conserved late‐stage transcriptional architecture linked to tissue remodeling and inflammatory signaling.

Regulatory network analysis further highlighted partially shared upstream transcriptional factors, such as HMGA1, NFKB1, and JUNB. JUNB and NFKB1 activity were preferentially associated with SASPs and inflammatory pathways, and HMGA1 with nucleolar stress and ribosomal activity; these regulators exhibited context‐dependent activity rather than uniform control across subtypes. Additionally, *LAMC2*, an ECM‐related gene shared across both senescence types, was predicted to be an NFKB1 target specifically in primary senescence and may contribute to inflammation and tissue remodeling‐associated programs. Given the inferential nature of SCENIC‐based network reconstruction, these regulatory relationships should be interpreted as putative associations that require future functional validation.

To ensure that shared transcriptional signatures were not driven by dataset separation or batch‐specific effects, we additionally performed integrated analysis across all experimental conditions (QUI, IR, QCMT, and SCMT). Shared markers remained enriched in senescent clusters following integration, supporting the robustness of the conserved transcriptional module across experimental contexts (Figure S6).

Our study highlights the importance of subclassifying SnCs into primary and secondary contexts when interpreting heterogeneity in senescence. Broad elimination of SnCs may overlook subtype‐specific biological functions, including context‐dependent contributions to tissue remodeling or inflammatory amplification. The identification of subtype‐enriched genes such as *LTBP2* and *CXCL8*, alongside shared regulatory networks involving HMGA1, NFKB1, and JUNB, provides a framework for future mechanistic studies aimed at understanding how distinct senescence states influence age‐related kidney pathology.

Nonetheless, several questions remain. Our conclusions are primarily based on transcriptomic inference, and although spatial transcriptomics analyses of young versus aged mouse kidneys and human CKD samples and limited protein‐level analyses provide complementary support, functional validation of candidate regulators and marker genes will be necessary to establish causality. In addition, the molecular mechanisms governing transitions from intermediate to terminal senescence states, particularly in secondary senescence, remain to be elucidated. The enrichment of long noncoding RNAs (lncRNAs) in primary senescent cluster C5 suggests a potential regulatory layer (Table S7). Although the functional roles of lncRNAs such as *AC090579.1*, *PAX8‐AS1*, and *AL136234.1* in senescence are currently unknown, further investigation is required to determine whether these transcripts actively contribute to metabolic remodeling or reflect condition‐specific transcriptional features (Grossi et al. 2024, 2025; Han 2023).

In summary, this study provides a single‐cell–resolved framework distinguishing primary and secondary senescence while defining a conserved transcriptional core shared across both contexts. These findings establish a descriptive foundation for future functional and translational studies aimed at selectively modulating primary and secondary senescent subtypes‐associated tissue remodeling and inflammation in kidney aging and disease.

## 4 Methods

### 4.1 10× Single‐Cell RNA‐Seq Library Preparation

Approximately 2.0 × 10^4^ cells were used for 10× scRNA‐seq. A 3′ single‐cell RNA‐seq library was prepared per 10× protocol, and quality control of cDNA and libraries was performed using a Tapestation. Libraries were sequenced on Illumina sequencers at the University of California, Davis, following the manufacturer's protocols. For each experimental condition (QUI, IR, QCMT, and SCMT), two independent biological replicates were generated and sequenced as independent libraries.

### 4.2 Real Time‐Quantitative Polymerase Chain Reaction (RT‐qPCR)

Total RNA was extracted from cells using a Direct‐zol RNA Miniprep Kit (Zymo Research, Irvine, CA, USA; R2050). RNA was reverse‐transcribed using the High‐Capacity cDNA Reverse Transcription Kit (Applied Biosystems, Waltham, MA, USA; 4368814). RT‐qPCR was performed on a QuantStudio 3 Real‐Time PCR System (Applied Biosystems) using the Power SYBR Green PCR Master Mix (Applied Biosystems; 4368706). The *β‐actin* gene was used as a housekeeping control. The primer sequences used in this experiment, including those for *CDKN2A* , *CDKN1A* , *IL‐1A*, *LMNB1* , *TNF‐α*, *IL‐6*, *IL‐8*, and *β‐actin*, are listed in Table S14.

### 4.3 Conditioned Media (CM) Treatment

Human renal epithelial cells (ATCC; PCS‐400‐011) were cultured in Renal Epithelial Cell Basal Medium (ATCC; PCS‐400‐030) supplemented with the Renal Epithelial Cell Growth Kit (ATCC; PCS‐400‐040), which maintains the cultures at a final serum concentration of 0.5% and incubated at 37°C in 10% CO_2_ and 3% O_2_. Collected and centrifuged CM were filtered through a 0.45‐μm syringe filter (Acrodisc). It was then mixed with Dulbecco's Modified Eagle Medium (DMEM) containing fetal bovine serum (FBS), resulting in CM with a final FBS concentration of 0.01%. Proliferating renal epithelial cells were exposed to this CM for 7 days to generate secondary SnCs and assess paracrine senescence.

### 4.4 Induction of Senescence via Exposure to Ionizing Radiation

Proliferating renal epithelial cells were subjected to ionizing radiation (10 Gy X‐ray) to trigger primary senescence. These primary SnCs were maintained for 10 days to allow full development of the senescent phenotype, while quiescent cells were cultured in 0.01% serum for 3 days. Following this, the cells were washed with phosphate‐buffered saline (Thermo Fisher Scientific, Waltham, MA; #10010‐023) and then cultured in serum‐ and phenol red‐free DMEM (Thermo Fisher Scientific; #21063‐029). After 48 h, the CM was collected.

### 4.5 Senescence‐Associated β‐Galactosidase (SA‐β‐Gal) Staining

SA‐β‐gal staining was conducted using a Senescence β‐Galactosidase Staining kit (Cell Signaling Technology, Danvers, MA, USA; 9860). SnCs were stained blue and visualized under a light microscope at 20× and 40×. After nuclear counterstaining with 4′,6‐diamidino‐2‐phenylindole (DAPI; EMD Millipore; 90229), the total number of cells was counted in ten randomly selected fields per culture dish. The percentage of SA‐β‐gal (+) cells was then quantified using ImageJ software. In each experiment, approximately 100–150 cells were analyzed.

### 4.6 5‐Ethynyl‐2′‐Deoxyuridine (EdU) Cell Proliferation Assay

Cell proliferation was assessed using Click‐iT EdU Alexa Fluor 488 imaging kits (Invitrogen; C10337). Primary human renal epithelial cells were incubated in DMEM with 10 μM EdU labeling solution for 24 h. Proliferation was quantified by determining the ratio of EdU‐stained nuclei to Hoechst‐positive cells in randomly selected fields.

### 4.7 Immunofluorescence Staining

Monolayer cultures of human renal epithelial cells were fixed with 4% paraformaldehyde (Biosesang, Gyeonggi‐do, Korea; P2031) and permeabilized with 0.5% Triton X‐100 (Biosesang; PR2294‐100‐74) for 20 min at room temperature. To minimize nonspecific binding, cells were blocked with 5% normal goat serum for 1 h, followed by overnight incubation at 4°C with a primary antibody against HMGB1 (1:100; Abcam, ab18256). For secondary detection, cells were incubated with Alexa Fluor 594‐conjugated goat anti‐rabbit IgG (H&L) (1:1000; Life Technologies, A‐11012) at room temperature. Nuclei were counterstained with 4′,6‐diamidino‐2‐phenylindole (DAPI; EMD Millipore; 90,229). Fluorescence images of nuclear HMGB1 were acquired using a fluorescence microscope (Carl Zeiss, USA).

### 4.8 Enzyme‐Linked Immunosorbent Assay (ELISA)

Secreted IL‐6 in conditioned media (CM) was quantified using a bead‐based ELISA kit (R&D Systems; HS600C), and human LTBP2 and IL‐8 (CXCL8) were measured by ELISA (Abcam; ab313988 and ab214030) following the manufacturers' instructions. Cells were seeded in 6‐well plates (65,000 cells/well; *n* = 6 per group), and CM and whole‐cell lysates were collected from independent culture plates (not paired). Lysates were prepared in RIPA buffer, and all samples were diluted using the kit‐provided diluent. CM measurements were normalized to cell number at harvest, whereas lysate inputs were normalized by total protein concentration prior to loading, and concentrations were interpolated from standard curves within the assay ranges (LTBP2: 17.5–5000 pg/mL; IL‐8: 3.91–250 pg/mL). ROUT‐identified outliers (Q = 1%) and values below the lower limit of quantitation (LLOQ) were excluded from both data display and statistical analysis.

### 4.9 Single‐Cell RNA‐Seq Analysis (scRNA‐Seq)

Raw sequencing reads were evaluated using FastQC for quality assessment. Raw sequencing data were processed using the Cell Ranger pipeline (10× Genomics). The Illumina sequencing files (BCL) were demultiplexed using the CellRanger mkfastq function. The reads were aligned to the GRCh38 reference genome, and count matrices were generated for each sample. For each condition, FASTQ files from two independent biological replicates were jointly processed using Cell Ranger count to generate condition‐specific feature–barcode matrices, thereby maximizing cellular coverage for downstream identification of rare senescent subpopulations. Count matrices were analyzed using the Seurat package (v4.4.0) in R (v4.3.3) (Stuart et al. 2019). Cells with poor‐quality metrics were filtered based on mitochondrial gene expression > 30% (percentage) or insufficient reads (nFeature_RNA < 500 or nCount_RNA < 500). After filtering, the primary senescence dataset included 34,740 cells, including 14,263 X‐ray irradiation‐induced senescent cells (IR) and 20,477 quiescent cells (QUI). The secondary senescence dataset included 39,916 cells: 20,940 cells treated with CM from SnCs (SCMT) and 18,976 cells treated with CM from quiescent cells (QCMT). The processed datasets were retained for downstream analyses. Each condition‐level matrix was normalized independently using SCTransform, regressing out mitochondrial gene percentages and unique molecular identifiers (UMI) counts. The top 3000 highly variable genes (HVGs) identified using FindVariableFeatures were used as integration features for downstream PCA and Harmony‐based alignment. Harmony (v1.2.3) was applied to SCTransform‐based PCA embeddings to align shared cell states across conditions for visualization and clustering. Dimensionality reduction and neighbor graph construction were based on Harmony‐corrected principal components. Clusters were identified through unsupervised clustering at a resolution of 0.3 using FindNeighbors and FindClusters. Cluster visualization was achieved using UMAP, and gene expression was visualized using the scCustomize package, with cluster annotations refined using the Rename_Clusters function in R.

### 4.10 Identification of Differentially Expressed Genes (DEGs)

DEGs were identified per cluster using Seurat's FindAllMarkers with Bonferroni correction for multiple testing based on the total number of tested genes. Genes with an absolute log_2_ fold change (|log_2_FC|) ≥ 0.25 and expressed in at least 25% of cells were considered significant. The identified DEGs for each cluster were analyzed using the DAVID database to determine the enriched Kyoto Encyclopedia of Genes and Genomes (KEGG) pathways and gene ontology (GO) terms (Huang et al. 2007). These analyses provided insights into the functional characteristics and molecular signatures associated with each subtype of SnCs. To identify common and specific genes, we compared DEGs between primary and secondary SnC datasets. Common genes were selected by overlapping DEGs and filtering based on fold change, statistical significance, and pseudotime expression trends to ensure robust characterization of shared molecular signatures. Specific genes were selected from unique DEGs in the QUI/IR and QCMT/SCMT datasets based on expression patterns, association with senescence, the SASPs, and relevance to renal function (Tables S15–S18) (Li et al. 2024).

### 4.11 Trajectory and Lineage Analysis

Lineage and pseudotemporal ordering of cells were inferred using Slingshot (v2.8.0) on UMAP dimensions (Street et al. 2018). Monocle3 (v1.3.1) was used to evaluate the pseudotime prediction generated by Slingshot (Trapnell et al. 2014). To identify genes dynamically expressed along pseudotime, the fitGAM() and associationTest() functions in the tradeSeq (v1.14.0) were utilized (Van den Berge et al. 2020). Genes with significant associations along pseudotime (FDR‐adjusted *p‐values* < 0.05) were filtered, and the top 500 DEGs were selected by ranking significant genes according to the Wald statistic (waldStat). Expression trends across pseudotime were predicted with predictSmooth(). Hierarchical clustering was applied to group genes with similar expression patterns and visualized with ComplexHeatmap (v2.16.0).

### 4.12 GO Enrichment Analysis

GO enrichment analysis was performed via the DAVID database for both DEGs and pseudotime‐derived gene clusters. Bar plots of enriched GO terms (*p‐values* < 0.05) were generated.

### 4.13 Gene Set Variation Analysis (GSVA)

GSVA (v1.48.3) was conducted to calculate enrichment scores for predefined gene sets and identify pathway alterations across primary and secondary SnC subclusters (Hänzelmann et al. 2013). Expression matrices were used to compute enrichment scores, providing pathway‐level insights into SnC subclusters.

### 4.14 Gene Set Enrichment Analysis (GSEA)

GSEA was conducted on normalized expression matrices to evaluate molecular pathways in fully senescent clusters, using 1000 phenotype‐based permutations (Subramanian et al. 2005).

### 4.15 Regulatory Network Analysis

Transcriptional regulatory networks were analyzed using pySCENIC (v.0.12.1) to infer transcription factor (TF) activity in primary and secondary senescence datasets (Van de Sande et al. 2020). Raw counts of HVGs were used to construct co‐expression modules, which were screened using RcisTarget with cis‐regulatory motifs from human motif databases (hg38_10kbp_up_10kbp_down_full_tx_v10_clust.genes_vs_motifs.rankings.feather) to identify candidate regulons. TFs and their predicted target genes, referred to as regulons, were assessed for regulon activity at single‐cell resolution. The regulon‐enriched cell populations were detected using the AUCell package. The resulting TF–target gene pairs were used to construct a regulatory network, which was visualized using Cytoscape (v3.10.3) (Shannon et al. 2003).

### 4.16 Spatial Transcriptomics

To demonstrate biological relevance, key genes were validated in aged kidney tissue using public spatial transcriptomics datasets from the Gene Expression Omnibus (GEO) (GSE252772). Mouse kidneys were collected at embryos (E16.5), newborns (P0), 3 weeks (W3), 12 weeks (W12), 52 weeks (W52), and 92 weeks (W92), as described in the original GEO dataset. For the present study, spatial transcriptomic data from postnatal stages (W3 and W92) were used for downstream analyses. Individual Visium datasets, preprocessed with SCTransform normalization, were merged into a single Seurat object. Batch effects across slides were corrected using Harmony, specifying sample identity (sample_id) as the batch variable to account for technical variation. Neighbor graph construction, clustering, and UMAP embedding were then performed using the Harmony‐corrected embeddings. Spatial gene expression maps were generated using the SpatialFeaturePlot function.

### 4.17 Kidney Precision Medicine Project (KPMP) Kidney Tissue Atlas Analysis

To assess clinical relevance in human kidney disease, spatial expression patterns were examined using the Kidney Precision Medicine Project (KPMP) Kidney Tissue Atlas (https://atlas.kpmp.org/). Gene expression maps were queried for healthy reference kidneys and CKD samples. Gene sets evaluated included primary senescence–specific DEGs, secondary senescence–specific DEGs, and common DEGs shared between primary and secondary senescence, as defined from our in vitro datasets. For each gene, spatial expression was visualized on the corresponding histological section using the Atlas viewer, and representative regions were selected for figure display (Figures S3 and S5). In CKD samples, fibrosis‐enriched regions of interest (ROIs) were operationally defined based on spatial co‐expression of ACTA2 and COL1A1, and expression patterns of selected genes from these sets were assessed within the ROI relative to surrounding tissue and healthy reference samples. Color scale bars shown in each panel correspond to the gene expression intensity scales provided by the KPMP viewer for the queried gene. This KPMP analysis was used for qualitative spatial validation/visualization rather than formal differential expression testing within the KPMP dataset.

### 4.18 Quantification and Statistical Analysis

Differential gene expression and pathway enrichment analyses were performed in R, with multiple testing correction by the Bonferroni method. GO term enrichment was conducted using DAVID with Fisher's Exact Test and Benjamini–Hochberg correction. Data visualization, including UMAP, violin, dot, heatmap, and network plots, was performed using ggplot2, scCustomize, ComplexHeatmap (v2.16.0), and Cytoscape (v3.10.3). Statistical significance thresholds were defined as *p < 0.05, **p < 0.01, ***p < 0.001, and ****p < 0.0001.

## Author Contributions

D.‐H.J. designed the experiments, performed in vitro assays, analyzed the data, and wrote the manuscript. E.S. performed single‐cell RNA sequencing analyses and wrote the manuscript. J.‐W.S., S.K., S.C., and H.J.K. integrated the studies, analyzed the data, and reviewed and edited the manuscript. Y.K. assisted with scRNA‐seq. T‐.H.G. contributed to figure generation using the KPMP Kidney Tissue Atlas. O.H.J. conceived the idea, planned and directed the studies, generated scRNA‐seq data, and wrote the manuscript. Review and editing were carried out by all authors.

## Funding

This work was supported by the National Research Foundation of Korea [Grant No. RS2024‐00340798 and RS‐2023‐00220894] and POSCO TJ Park Foundation.

## Conflicts of Interest

The authors declare no conflicts of interest.

## Supporting information


**Figure S1:** Single‐cell characterization of primary senescence in human renal epithelial cells. (A) Representative immunofluorescence and histochemical images of DAPI (nuclei), EdU incorporation (proliferation), and SA‐β‐gal staining in quiescent control cells (QUI) and irradiated primary renal epithelial cells (IR). Scale bar, 150 μm. (B) Quantification of EdU‐positive cells and (C) SA‐β‐gal–positive cells (%) in QUI and IR conditions. Percentages were calculated as EdU (+) or SA‐β‐gal (+) cells divided by total DAPI (+) nuclei for each condition; each dot represents an independent replicate (QUI, *n* = 5; IR, *n* = 5). (D) UMAP visualization of the primary senescence scRNA‐seq dataset showing eleven transcriptionally distinct clusters. (E) Cell cycle phase distribution across clusters. Stacked bar plot showing the proportion of cells in each phase per cluster. Clusters were categorized as non‐senescent (C4 and C9), intermediate (C0, C1, C3, and C7), or fully senescent (C5, C6, and C8) based on transcriptomic features. (F) Heatmap of pathway activity differences (GO, KEGG) across cell subclusters and scored via gene set variation analysis. Pathway scores are normalized as a *Z*‐score (blue, low; red, high). (G) Heatmap of SASP‐related gene expression (SASP Atlas gene set) across primary senescence subtypes. (H) Functional enrichment analysis of genes upregulated in cluster C8 among the top 500 pseudotime‐associated genes identified by tradeSeq along lineage 3. Enrichment analysis was performed using DAVID, and significantly enriched terms (*p* < 0.05) are shown, ranked by GeneRatio (Count/ListTotal, where Count is the number of input genes mapped to each term and ListTotal is the total number of input genes), and the top five terms are displayed.
**Figure S2:** Single‐cell characterization of secondary senescence in human renal epithelial cells. (A) Representative immunofluorescence images of HMGB1, EdU, and SA‐β‐gal staining in QUI conditioned media (CM)‐treated (QCMT) and IR‐CM‐treated (SCMT) cells. Scale bars, 75 μm. (B) Quantification of HMGB1‐positive cells (%) in QCMT and SCMT conditions (QCMT, *n* = 7; SCMT, *n* = 12). (C) Quantification of EdU‐positive cells (%) in QCMT and SCMT conditions. Percentages were calculated as EdU (+) cells divided by total DAPI (+) nuclei. Each dot represents an independent biological replicate (QCMT, *n* = 9; SCMT, *n* = 11). (D) Quantification of SA‐β‐gal–positive cells (%). Percentages were calculated as SA‐β‐gal (+) cells divided by total DAPI (+) nuclei for each condition; each dot represents an independent replicate (QCMT, *n* = 6; SCMT, *n* = 6). (E) UMAP visualization revealed eight transcriptionally distinct clusters of secondary senescent cells. (F) Cell cycle phase distribution across clusters. Stacked bar plot showing the proportion of cells in each phase per cluster. Clusters were categorized as non‐senescent (C2 and C6), intermediate (C4, C0, and C1), or fully senescent (C7, C3, and C5) based on transcriptomic features. (G) Heatmap of pathway activities (GO and KEGG) scored using gene set variation analysis. Pathway scores are *Z*‐score‐normalized (blue = low, red = high), highlighting renal, collagen, and autophagy‐related programs. (H) Heatmap of expression levels for SASP‐related genes from the SASP Atlas across secondary senescence subtypes. (I) Functional enrichment analysis of genes upregulated in cluster C3 among the top 500 pseudotime‐associated genes identified by tradeSeq along lineage 4. Enrichment analysis was performed using DAVID (GO, KEGG, and Reactome), and significantly enriched terms (*p* < 0.05) are shown, ranked by GeneRatio (Count/ListTotal, where Count is the number of input genes mapped to each term and ListTotal is the total number of input genes), and the top five terms are displayed.
**Figure S3:** Spatial comparison of primary and secondary senescence–associated DEG expression between healthy controls and CKD human kidney tissues (KPMP Kidney Tissue Atlas). (A) Representative spatial transcriptomic maps from the KPMP Kidney Tissue Atlas comparing healthy controls (left) and patients with chronic kidney disease (CKD) (right). Genes are grouped into primary senescence–specific DEGs (top) and secondary senescence–specific DEGs (bottom), as defined from our single‐cell datasets. In CKD samples, arrows and dashed outlines indicate regions operationally defined as fibrotic based on spatial co‐expression of *ACTA2* and *COL1A1*. Multiple subtype‐associated DEGs display increased or spatially enriched expression within these fibrotic areas compared with healthy reference tissues. The color scale shown in each panel corresponds to the normalized gene expression intensity provided by the KPMP spatial viewer for the queried gene.
**Figure S4:** ELISA‐based validation of LTBP2 and CXCL8 protein levels in conditioned media (CM) and cell lysates across senescence models. (A) LTBP2 in CM (pg/mL per 10^4^ cells), QUI vs. IR (filtered n: QUI, *n* = 6; IR, *n* = 4). (B) CXCL8 in cell lysates (protein‐normalized), QUI vs. IR (filtered n: QUI, *n* = 5; IR, *n* = 6). (C) LTBP2 in CM (pg/mL per 10^4^ cells), QCMT vs. SCMT (filtered n: QCMT, *n* = 6; SCMT, *n* = 5). (D) CXCL8 in cell lysates (protein‐normalized), QCMT vs. SCMT (filtered n: QCMT, *n* = 6; SCMT, *n* = 5). CM concentrations were normalized to cell number and are reported as pg/mL per 10^4^ cells. Lysate samples were loaded at equal total protein input per well (protein‐normalized) prior to ELISA. ROUT outliers identified in GraphPad Prism (Q = 1%) and values below the lower limit of quantitation (< LLOQ) were excluded prior to plotting and statistical analysis. Initial sample sizes were QUI (*n* = 6), IR (*n* = 6), QCMT (*n* = 6), and SCMT (*n* = 5). Each dot represents one biological sample. For each panel, statistical significance was assessed using an unpaired two‐tailed Welch's *t*‐test. Significance: *p < 0.05, **p < 0.01, ***p < 0.001.
**Figure S5:** Spatial comparison of common DEGs shared between primary and secondary senescence in human CKD kidney tissues (KPMP Kidney Tissue Atlas). (A) Representative spatial transcriptomic maps from the KPMP Kidney Tissue Atlas comparing healthy reference kidneys (left) and CKD patient tissues (right). Genes shown are common DEGs shared between primary and secondary senescence, as defined from our single‐cell analyses datasets (e.g., *IGFBP5*, *LAMC2*, *KRT7*, *ITGB6*, *TFAP2B*). In CKD samples, arrows and dashed outlines indicate regions operationally defined as fibrotic based on spatial co‐expression of *ACTA2* and *COL1A1*. The color scale bar shown in each panel corresponds to the gene expression intensity scale provided by the KPMP viewer for the queried gene.
**Figure S6:** Integrated analysis of primary and secondary senescence datasets. (A) Uniform manifold approximation and projection (UMAP) visualization of the integrated scRNA‐seq dataset combining QUI, IR, QCMT, and SCMT conditions, identifying eight transcriptionally distinct clusters (left). Bar plot (right) shows the proportion of cells from each condition within each cluster. (B) UMAP colored by assigned cell cycle phase (G1, G2M, S) (left) and stacked bar plot (right) showing the relative distribution of cell cycle phases across clusters. (C) Feature plots showing expression of proliferation‐related genes (MKI67, CCNA2, TOP2A, and CDKN3) and senescence‐associated genes (CDKN2A, CDKN1A, NFKB1, and TP53) across the integrated dataset. (D) Boxplots of normalized SASP‐related gene set scores (SAUL_SEN_MAYO signature) across clusters. Statistical significance was determined using the Kruskal–Wallis test followed by Wilcoxon rank‐sum test for pairwise comparison (adjusted *p*‐values ***p < 2.2 × 10–16). (E) Dot plots showing expression patterns of five commonly upregulated genes shared between primary and secondary senescence in the integrated dataset. Dot color represents normalized mean expression levels, and dot size indicates the percentage of cells expressing the respective genes in each cluster.

**Table S1:** All DEG list of QUI/IR and QCMT/SCMT dataset.
**Table S2:** The significant GO and KEGG pathways selected for each cluster in QUI/IR and QCMT/SCMT datasets.
**Table S3:** Tradeseq heatmap associated gene list and pathway analysis for QUI/IR and QCMT/SCMT datasets.
**Table S4:** GO and KEGG pathways for distinguishing non‐senescent and senescence‐resistant cells.
**Table S5:** Specific gene list observed in QUI/IR and QCMT/SCMT.
**Table S6:** Specific gene list observed in QUI/IR (clusters 5,6,8) and QCMT/SCMT (clusters 3,5,7).
**Table S7:** Analysis of the relationships between senescence, the SASPs, and renal pathways, and identification of GO and KEGG pathways involving specific genes in primary and secondary SnCs.
**Table S8:** Common gene list observed in QUI/IR (clusters 5, 6, 8) and QCMT/SCMT (clusters 3,5,7).
**Table S9:** Analysis of the relationships between senescence, the SASPs, and renal pathways, and identification of GO and KEGG pathways involving common genes in primary and secondary SnCs.
**Table S10:** QUI/IR and QCMT/SCMT SCENIC.
**Table S11:** Pathways related to HMGA1, NFKB1, and JUNB‐modulated DEGs in QUI/IR and QCMT/SCMT.
**Table S12:** HMGA1, NFKB1, and JUNB ‐modulated DEGs in QUI/IR and QCMT/SCMT.
**Table S13:** All DEG lists of QUI/IR and QCMT/SCMT integrated datasets.
**Table S14:** Primer Sequences Used in RT‐qPCR Analysis.
**Table S15:** Senescence‐related gene sets from known databases or studies.
**Table S16:** SASP atlas.
**Table S17:** Gene list associated with renal disease and related pathways from known databases or studies.
**Table S18:** Renal‐related upregulated GO and KEGG pathways in QUI/IR and QCMT/SCMT datasets.

## Data Availability

The datasets generated and analyzed during the current study are available in the NCBI Gene Expression Omnibus (GEO) repository under accession number GSE292762 [https://www.ncbi.nlm.nih.gov/geo/query/acc.cgi?acc=GSE292762]. All other data supporting the findings of this study are included in this published article and its Supporting Information files. The R code scripts used to perform the main steps of the analysis of the scRNA‐seq data are available at https://github.com/ehshim/20250404_GEO‐GSE292762.
