# Supplementary material for: Transcriptional Profiling at Single‐Cell Resolution Reveals Diversity and Regulatory Networks of Primary and Secondary Senescent Cells
Source: Aging Cell. 2026 May 18;25(5):e70540. doi: 10.1111/acel.70540 (PMC13182272; doi:10.1111/acel.70540)
Supplement: Supplementary file 1 — Figure S1: Single‐cell characterization of primary senescence in human renal epithelial cells. (A) Representative immunofluorescence and histochemical images of DAPI (nuclei), EdU incorporation (proliferation), and SA‐β‐gal staining in quiescent control cells (QUI) and irradiated primary renal epithelial cells (IR). Scale bar, 150 μm. (B) Quantification of EdU‐positive cells and (C) SA‐β‐gal–positive cells (%) in QUI and IR conditions. Percentages were calculated as EdU (+) or SA‐β‐gal (+) cells divided by total DAPI (+) nuclei for each condition; each dot represents an independent replicate (QUI, n = 5; IR, n = 5). (D) UMAP visualization of the primary senescence scRNA‐seq dataset showing eleven transcriptionally distinct clusters. (E) Cell cycle phase distribution across clusters. Stacked bar plot showing the proportion of cells in each phase per cluster. Clusters were categorized as non‐senescent (C4 and C9), intermediate (C0, C1, C3, and C7), or fully senescent (C5, C6, and C8) based on transcriptomic features. (F) Heatmap of pathway activity differences (GO, KEGG) across cell subclusters and scored via gene set variation analysis. Pathway scores are normalized as a Z‐score (blue, low; red, high). (G) Heatmap of SASP‐related gene expression (SASP Atlas gene set) across primary senescence subtypes. (H) Functional enrichment analysis of genes upregulated in cluster C8 among the top 500 pseudotime‐associated genes identified by tradeSeq along lineage 3. Enrichment analysis was performed using DAVID, and significantly enriched terms (p < 0.05) are shown, ranked by GeneRatio (Count/ListTotal, where Count is the number of input genes mapped to each term and ListTotal is the total number of input genes), and the top five terms are displayed. Figure S2: Single‐cell characterization of secondary senescence in human renal epithelial cells. (A) Representative immunofluorescence images of HMGB1, EdU, and SA‐β‐gal staining in QUI conditioned media (CM)‐treated (QCMT) and IR‐ [file ACEL-25-e70540-s002.docx]

**Supplementary Figures and Legends**


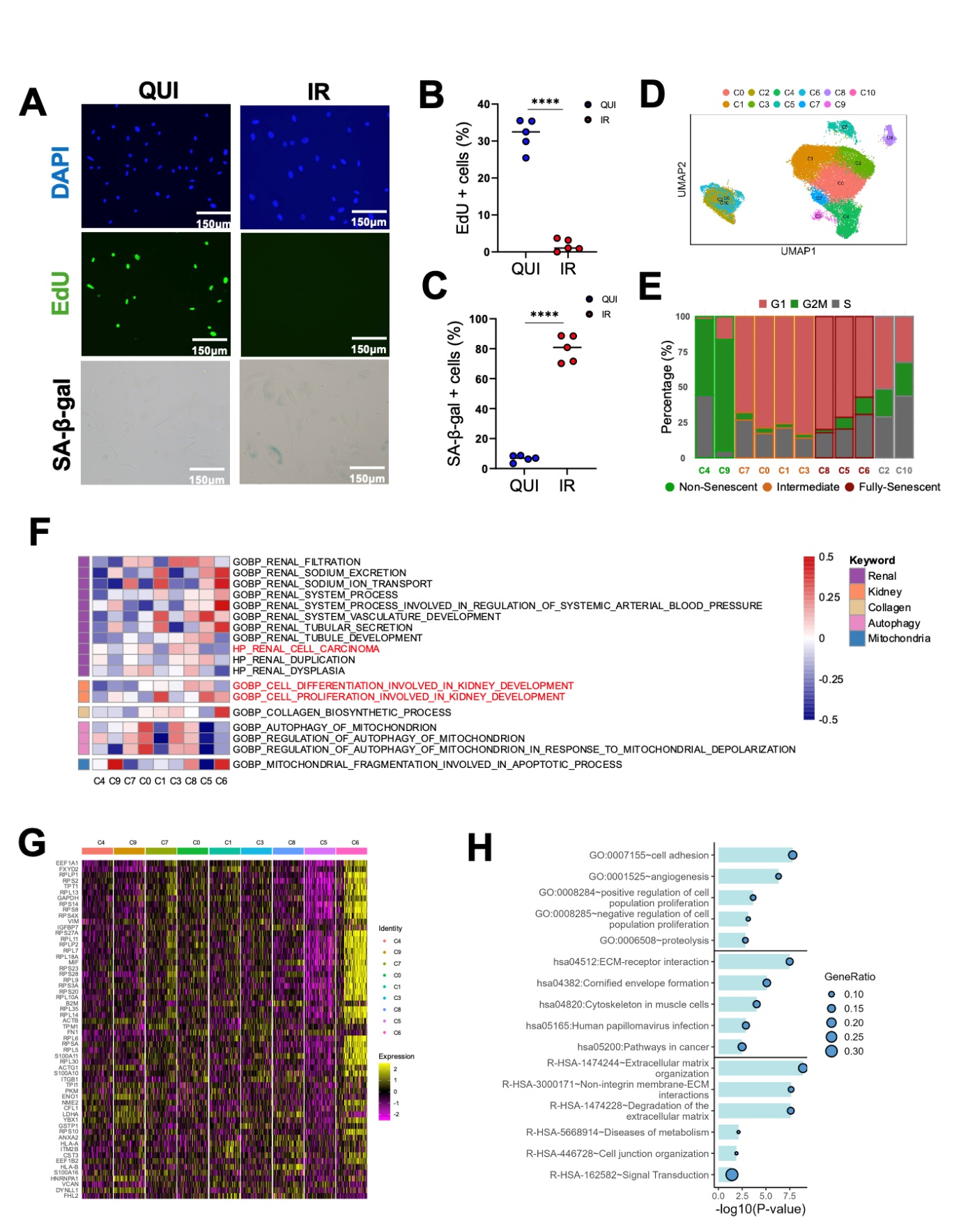


**Figure S1. Single-cell characterization of primary senescence in human renal epithelial cells. (A)** Representative immunofluorescence and histochemical images of DAPI (nuclei), EdU incorporation (proliferation), and SA-β-gal staining in quiescent control cells (QUI) and irradiated primary renal epithelial cells (IR). Scale bar, 150 μm. **(B)** Quantification of EdU-positive cells (%) and **(C)** SA-β-gal–positive cells (%) in QUI and IR conditions. Percentages were calculated as EdU (+) or SA-β-gal (+) cells divided by total DAPI (+) nuclei for each condition; each dot represents an independent replicate (QUI, n =5; IR, n=5). **(D)** UMAP visualization of the primary senescence scRNA-seq dataset showing eleven transcriptionally distinct clusters. **(E)** Cell cycle phase distribution across clusters. Stacked bar plot showing the proportion of cells in each phase per cluster. Clusters were categorized as non-senescent (C4 and C9), intermediate (C0, C1, C3, and C7), or fully senescent (C5, C6, and C8) based on transcriptomic features. **(F)** Heatmap of pathway activity differences (GO, KEGG) across cell subclusters and scored via gene set variation analysis. Pathway scores are normalized as a Z-score (blue, low; red, high). **(G)** Heatmap of SASP-related gene expression (SASP Atlas gene set) across primary senescence subtypes. **(H)** Functional enrichment analysis of genes upregulated in cluster C8 among the top 500 pseudotime-associated genes identified by tradeSeq along lineage 3. Enrichment analysis was performed using DAVID, and significantly enriched terms (*p* < 0.05) are shown, ranked by GeneRatio (Count/ListTotal, where Count is the number of input genes mapped to each term and ListTotal is the total number of input genes), and the top five terms are displayed.


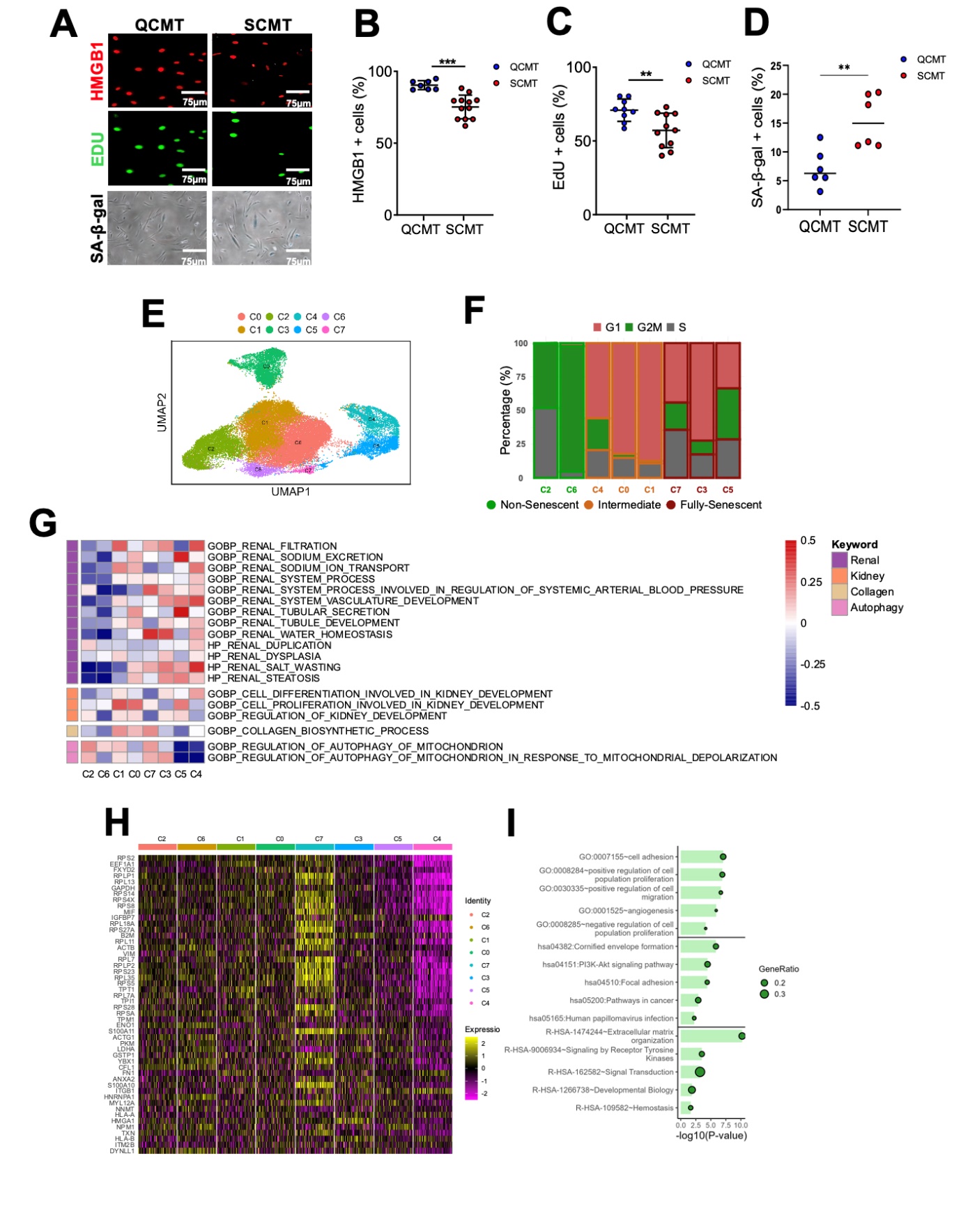


**Figure S2. Single-cell characterization of secondary senescence in human renal epithelial cells. (A)** Representative immunofluorescence images of HMGB1, EdU, and SA-β-gal staining in QUI conditioned media (CM)-treated (QCMT) and IR-CM-treated (SCMT) cells. Scale bars, 75 μm. **(B)** Quantification of HMGB1-positive cells (%) in QCMT and SCMT conditions (QCMT, n=7; SCMT, n=12). **(C)** Quantification of EdU-positive cells (%) in QCMT and SCMT conditions. Percentages were calculated as EdU (+) cells divided by total DAPI (+) nuclei. Each dot represents an independent biological replicate (QCMT, n=9; SCMT, n=11). **(D)** Quantification of SA-β-gal–positive cells (%). Percentages were calculated as SA-β-gal (+) cells divided by total DAPI (+) nuclei for each condition; each dot represents an independent replicate (QCMT, n = 6; SCMT, n = 6). **(E)** UMAP visualization revealed eight transcriptionally distinct clusters of secondary senescent cells. **(F)** Cell cycle phase distribution across clusters. Stacked bar plot showing the proportion of cells in each phase per cluster. Clusters were categorized as non-senescent (C2 and C6), intermediate (C4, C0, and C1), or fully senescent (C7, C3, and C5) based on transcriptomic features. **(G)** Heatmap of pathway activities (GO and KEGG) scored using gene set variation analysis. Pathway scores are Z-score-normalized (blue = low, red = high), highlighting renal, collagen, and autophagy-related programs. **(H)** Heatmap of expression levels for SASP-related genes from the SASP Atlas across secondary senescence subtypes. **(I)** Functional enrichment analysis of genes upregulated in cluster C3 among the top 500 pseudotime-associated genes identified by tradeSeq along lineage 4. Enrichment analysis was performed using DAVID (GO, KEGG, and Reactome), and significantly enriched terms (*p* < 0.05) are shown, ranked by GeneRatio (Count/ListTotal, where Count is the number of input genes mapped to each term and ListTotal is the total number of input genes), and the top five terms are displayed.


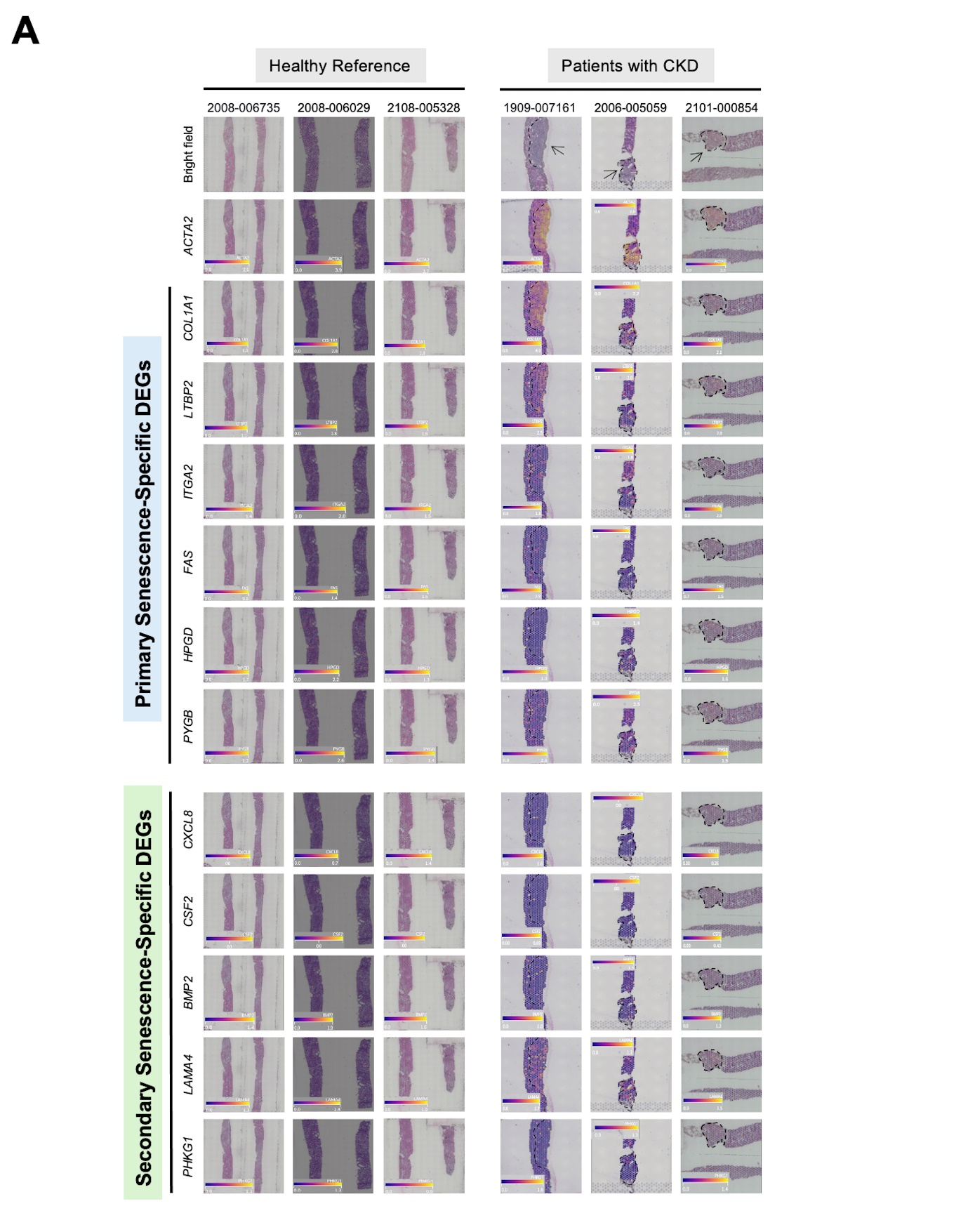


**Figure S3. Spatial comparison of primary and secondary senescence–associated DEG expression between healthy controls and CKD human kidney tissues (KPMP Kidney Tissue Atlas). (A)** Representative spatial transcriptomic maps from the KPMP Kidney Tissue Atlas comparing healthy controls (left) and patients with chronic kidney disease (CKD) (right). Genes are grouped into primary senescence–specific DEGs (top) and secondary senescence–specific DEGs (bottom), as defined from our single-cell datasets. In CKD samples, arrows and dashed outlines indicate regions operationally defined as fibrotic based on spatial co-expression of *ACTA2* and *COL1A1*. Multiple subtype-associated DEGs display increased or spatially enriched expression within these fibrotic areas compared with healthy reference tissues. The color scale shown in each panel corresponds to the normalized gene expression intensity provided by the KPMP spatial viewer for the queried gene.


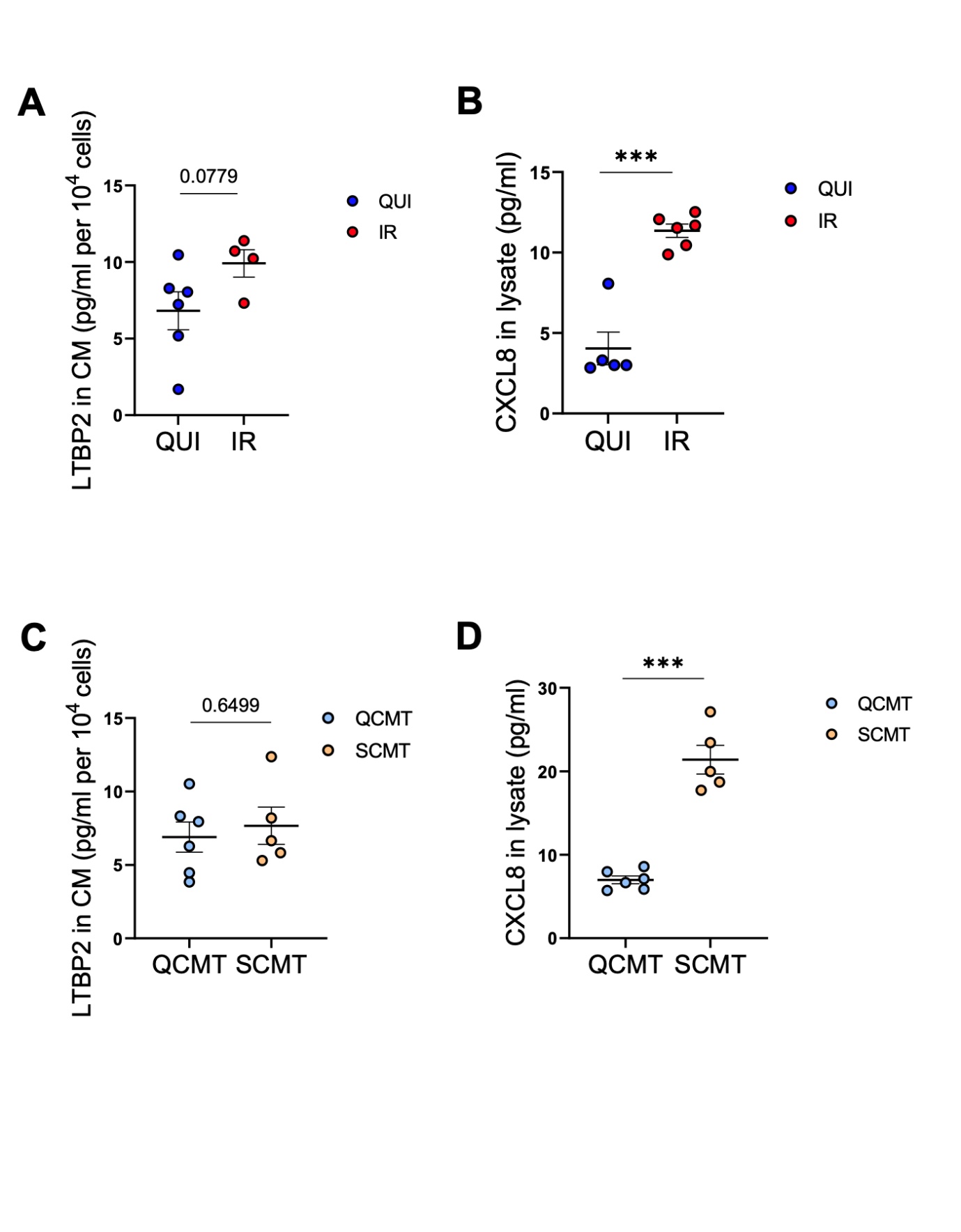


**Figure S4. ELISA-based validation of LTBP2 and CXCL8 protein levels in conditioned media (CM) and cell lysates across senescence models. (A)** LTBP2 in CM (pg/mL per 10⁴ cells), QUI vs IR (filtered n: QUI, n= 6; IR, n= 4). **(B)** CXCL8 in cell lysates (protein-normalized), QUI vs IR (filtered n: QUI, n= 5; IR, n= 6). **(C)** LTBP2 in CM (pg/mL per 10⁴ cells), QCMT vs SCMT (filtered n: QCMT, n= 6; SCMT, n= 5). **(D)** CXCL8 in cell lysates (protein-normalized), QCMT vs SCMT (filtered n: QCMT, n= 6; SCMT, n= 5). CM concentrations were normalized to cell number and are reported as pg/mL per 10⁴ cells. Lysate samples were loaded at equal total protein input per well (protein-normalized) prior to ELISA. ROUT outliers identified in GraphPad Prism (Q = 1%) and values below the lower limit of quantitation (<LLOQ) were excluded prior to plotting and statistical analysis. Initial sample sizes were QUI (n = 6), IR (n = 6), QCMT (n = 6), and SCMT (n = 5). Each dot represents one biological sample. For each panel, statistical significance was assessed using an unpaired two-tailed Welch’s t-test. Significance: **p* < 0.05, ***p* < 0.01, ****p* < 0.001.


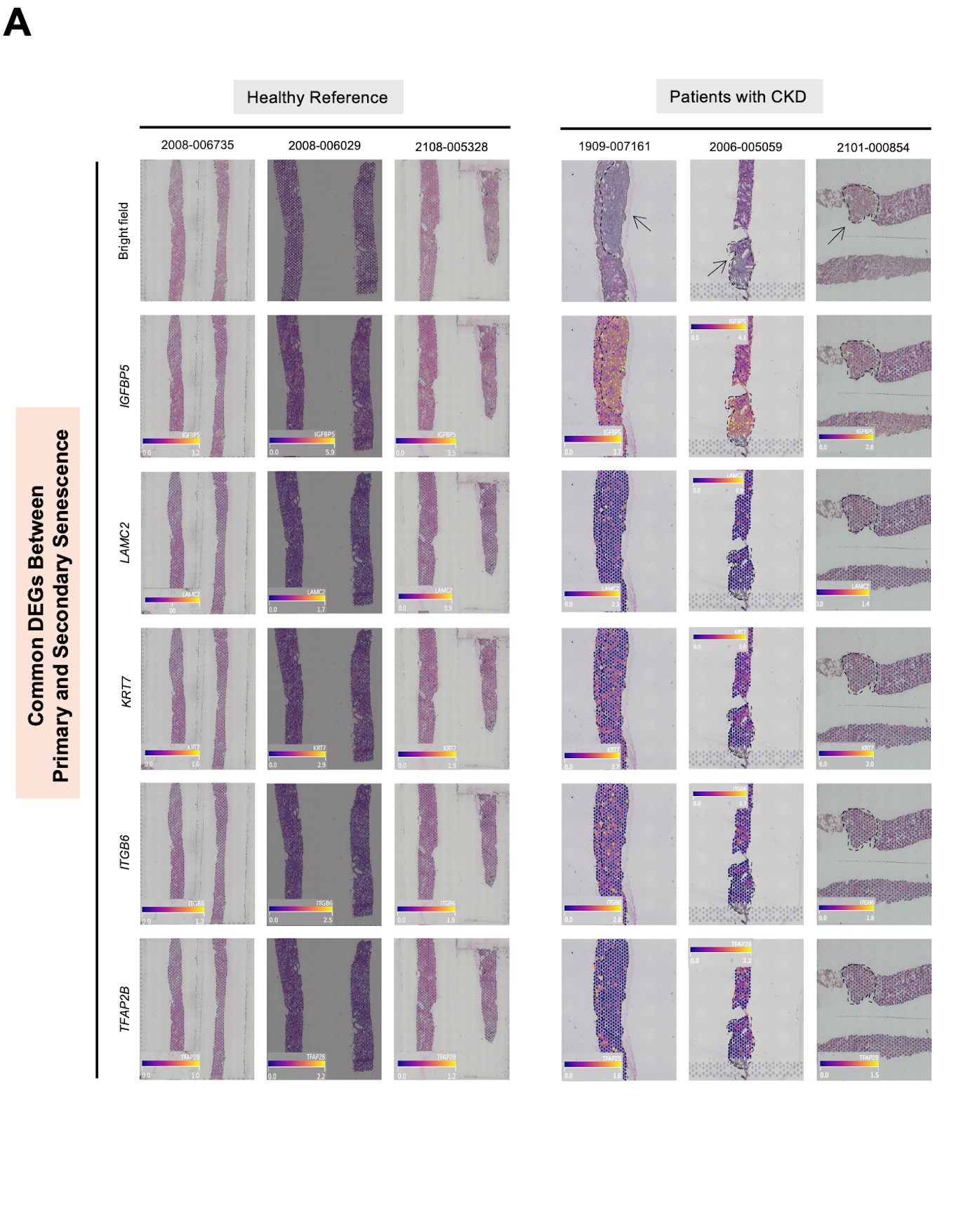


**Figure S5. Spatial comparison of common DEGs shared between primary and secondary senescence in human CKD kidney tissues (KPMP Kidney Tissue Atlas). (A)** Representative spatial transcriptomic maps from the KPMP Kidney Tissue Atlas comparing healthy reference kidneys (left) and CKD patient tissues (right). Genes shown are common DEGs shared between primary and secondary senescence, as defined from our single-cell analyses datasets (e.g., *IGFBP5*, *LAMC2*, *KRT7*, *ITGB6*, *TFAP2B*). In CKD samples, arrows and dashed outlines indicate regions operationally defined as fibrotic based on spatial co-expression of *ACTA2* and *COL1A1*. The color scale bar shown in each panel corresponds to the gene expression intensity scale provided by the KPMP viewer for the queried gene.


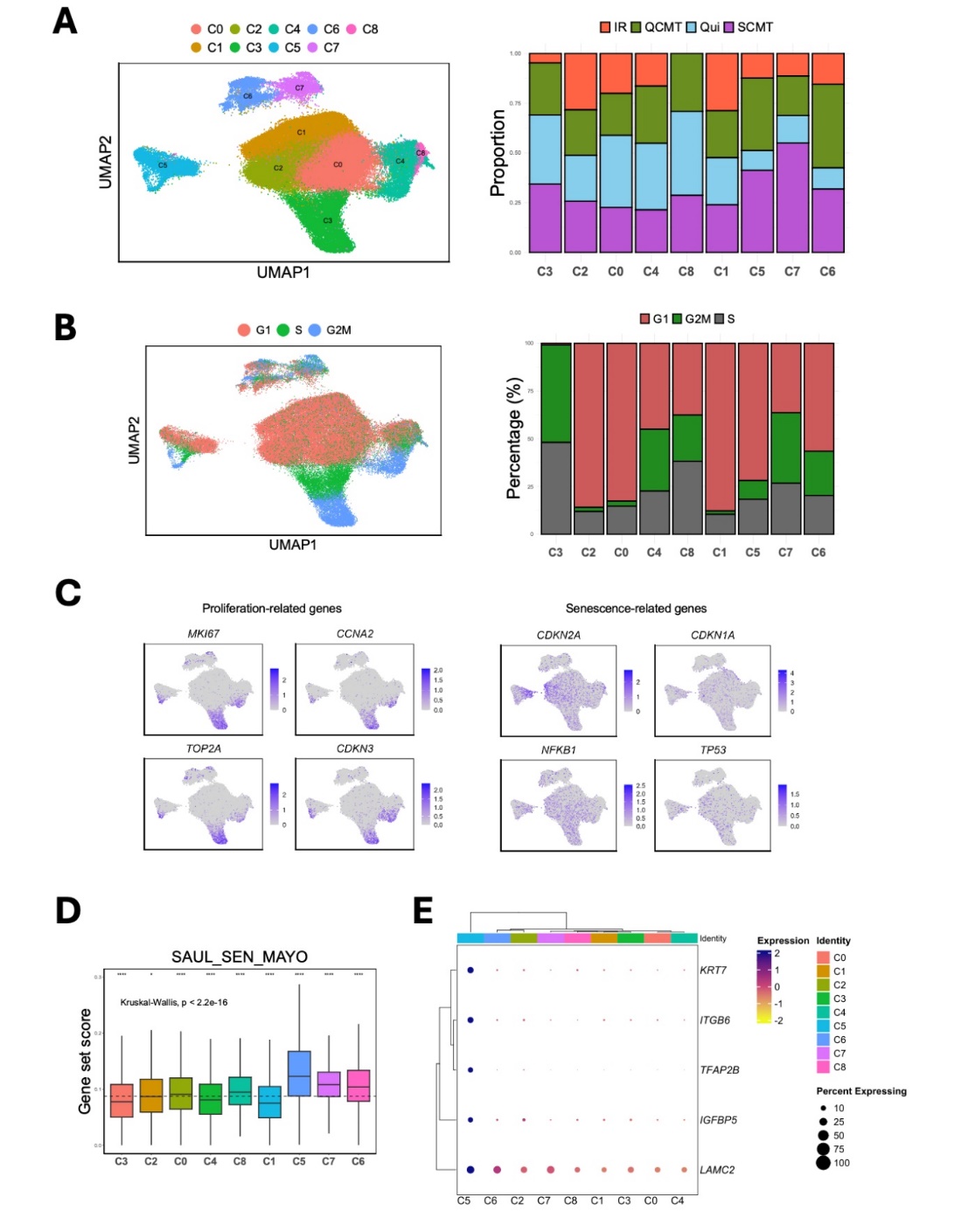


**Fig S6. Integrated analysis of primary and secondary senescence datasets. (A)** Uniform manifold approximation and projection (UMAP) visualization of the integrated scRNA-seq dataset combining QUI, IR, QCMT, and SCMT conditions, identifying eight transcriptionally distinct clusters (left). Bar plot (right) shows the proportion of cells from each condition within each cluster. **(B)** UMAP colored by assigned cell cycle phase (G1, G2M, S) (left) and stacked bar plot (right) showing the relative distribution of cell cycle phases across clusters. **(C)** Feature plots showing expression of proliferation-related genes (MKI67, CCNA2, TOP2A, CDKN3) and senescence-associated genes (CDKN2A, CDKN1A, NFKB1, TP53) across the integrated dataset. **(D)** Boxplots of normalized SASP-related gene set scores (SAUL_SEN_MAYO signature) across clusters. Statistical significance was determined using the Kruskal-Wallis test followed by Wilcoxon rank-sum test for pairwise comparison (adjusted p-values ***p < 2.2 × 10−16). **(E)** Dot plots showing expression patterns of five commonly upregulated genes shared between primary and secondary senescence in the integrated dataset. Dot color represents normalized mean expression levels, and dot size indicates the percentage of cells expressing the respective genes in each cluster.
